# Mobile health for chronic obstructive pulmonary disease: a bibliometric analysis based on integrated databases (2000–2025)

**DOI:** 10.3389/fmed.2026.1803815

**Published:** 2026-06-02

**Authors:** Yubei Liu, Xuetao Yang, Xuejun Liu, Rong Wang

**Affiliations:** 1 Dazhou Vocational College of Traditional Chinese Medicine, Dazhou, China; 2 Chengdu Xindu District Second People's Hospital, Chengdu, China

**Keywords:** bibliometrics, chronic disease management, clinical trial, COPD, mHealth, visualized analysis

## Abstract

**Background:**

Chronic obstructive pulmonary disease (COPD) is a major global health burden. Mobile health (mHealth) has created new possibilities for the monitoring, rehabilitation, and management of COPD, yet a dedicated bibliometric analysis of this field is still lacking. This study aimed to characterize the research landscape, assess the current status, and explore future developmental trends.

**Purpose:**

To map the research status and developmental trends of mHealth for COPD through bibliometric analysis and provide evidence-based guidance for future research.

**Methods:**

Publications indexed in the Science Citation Index Expanded of the Web of Science Core Collection and clinical trials indexed in PubMed were retrieved and screened for the period 2000–2025. CiteSpace and VOSviewer were used to analyze publication trends, leading journals, countries, institutions, authors, keywords, and cited references. The screening and reporting process followed the BIBLIO checklist.

**Results:**

After screening, 1,177 publications from the Web of Science Core Collection and 119 clinical trials from PubMed were included. Research output and annual total citations showed sustained growth over time. The *Journal of Medical Internet Research* ranked first in publication volume, whereas the *Journal of Telemedicine and Telecare* had the highest total citations. The United States was the most productive country, England ranked first in total citations, and Canada had the highest average citations per publication. The University of Toronto was the most productive institution, and Anne E. Holland and Michele Vitacca were the most prolific authors. Research hotspots mainly centered on COPD, telehealth, self-management, pulmonary rehabilitation, and quality of life. Remote monitoring and early warning, tele- and home-based rehabilitation, mHealth self-management support, and smart medication adherence management were identified as major functional components of mHealth. Artificial intelligence (AI), machine learning, and Internet of Things (IoT) technologies represented major emerging frontiers. For clinical research, future priorities included multicenter large-scale randomized controlled trials, integrated digital interventions for multimorbidity management, optimized remote strategies for exacerbation prevention and pulmonary rehabilitation, and further development of AI- and IoT-enabled personalized care.

**Conclusion:**

This study provides a comprehensive bibliometric overview of mHealth research in COPD. The findings highlight sustained growth, evolving research priorities, and rising academic impact in this field, while also revealing marked geographical disparities in research output. More robust, evidence-based, and context-adapted digital health strategies are needed to support the broader and more equitable development of this field.

## 1 Introduction

Chronic obstructive pulmonary disease (COPD), a progressive disease characterized by airflow limitation and chronic airway inflammation, remains a major global health burden (1, 2). Its progressive and fluctuating course requires comprehensive and continuous care strategies that extend beyond episodic clinical visits and integrate early identification, portable monitoring, rehabilitation support, long-term management, and patient-clinician communication. In this context, mobile health (mHealth) has emerged as a promising approach to supporting such comprehensive and continuous care (3–5). In this study, mHealth was defined as healthcare and public health interventions delivered or supported through mobile or portable digital devices, such as smartphones, tablets, wearable devices, portable monitoring devices, and related applications (5). Digital health was regarded as a broader umbrella term encompassing the use of digital technologies to support healthcare delivery and health management. Related terms, including eHealth, telehealth, telemedicine, remote monitoring, and telerehabilitation, were considered relevant but not identical concepts, as they differ in scope, technological focus, and mode of care delivery (6–8). Because this bibliometric analysis focused specifically on interventions supported by mobile or portable devices for COPD rather than digital health in general, mHealth was retained as the principal term throughout this manuscript.

Over the past 25 years, mHealth research in COPD has accumulated steadily and diversified substantially. Early studies mainly explored telecare and telehealth models, whereas more recent work has increasingly involved remote monitoring, telerehabilitation, self-management support, and adherence management (7, 8). These developments suggest that mHealth for COPD has evolved from relatively simple remote care models toward more integrated digital approaches that support monitoring, rehabilitation, self-management, and long-term disease management. However, despite this growing body of work, important challenges remain, including technical instability, limited acceptance, low digital literacy among older adults, and inadequate data privacy protection (9, 10). As mHealth research for COPD continues to expand and diversify, a structured overview is needed to depict its evolution, research structure, and emerging directions.

**Figure 1:**
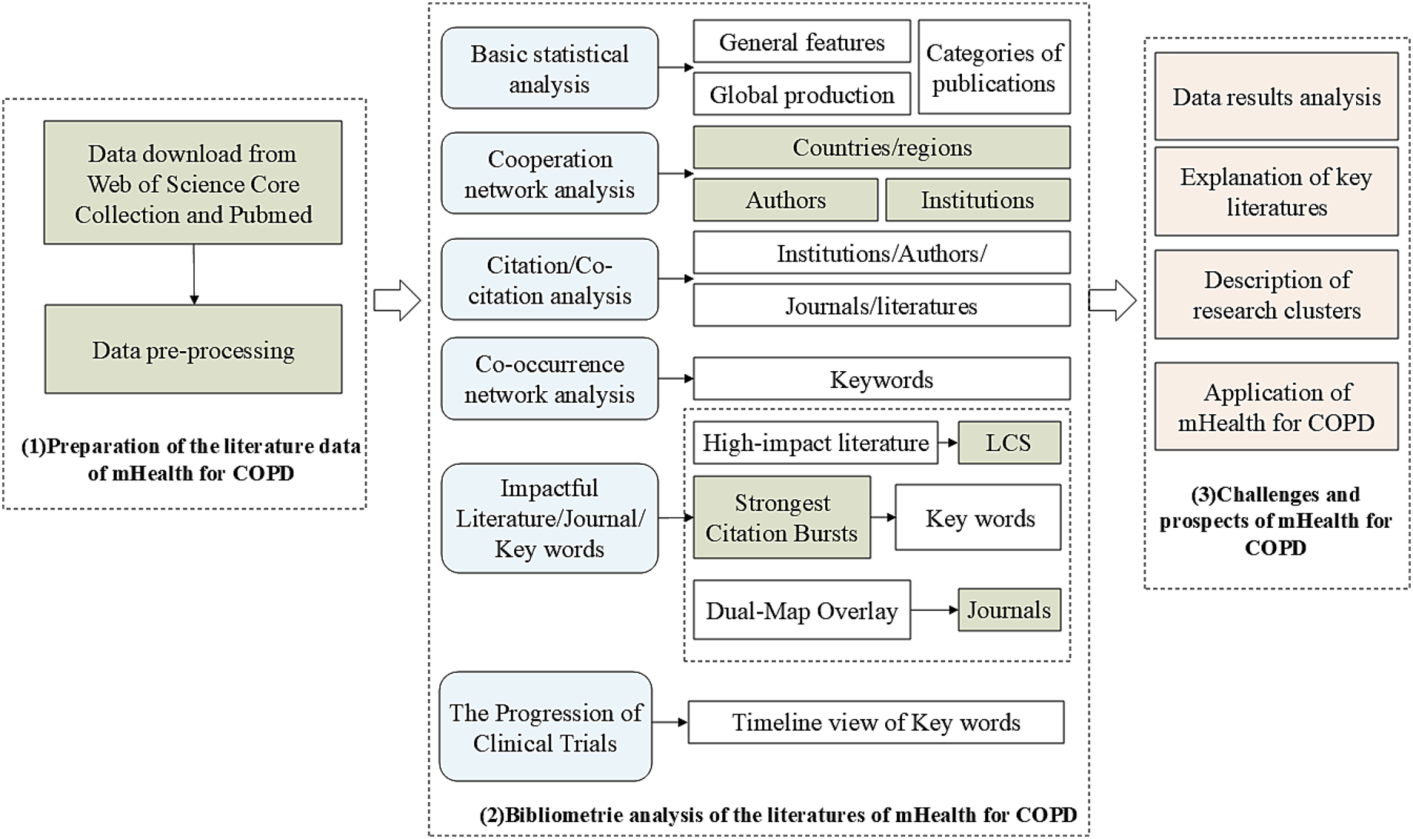
Analytical framework of the bibliometric analysis of mHealth research for COPD.

Bibliometrics is a method for identifying research patterns and trends through quantitative and qualitative analysis of published literature. By integrating information on publications, citations, collaborations, and keywords, bibliometrics helps clarify how a research field develops and how its major themes evolve (11). Previous reviews have provided valuable summaries of digital care approaches from the perspective of COPD management (12). Bibliometric approaches have also been used in narrower digital care topics related to COPD, such as e-health tools for inhalation therapy adherence (13). However, a dedicated bibliometric analysis that comprehensively examines publication trends, knowledge structure, collaboration patterns, research hotspots, and clinical trial development in mHealth for COPD is still lacking. Using PubMed and the Web of Science Core Collection (WoSCC), this study employed bibliometric tools to achieve four objectives (Figure 1). These objectives were to: (1) provide an overview of global research development in this field; (2) identify productive institutions, influential journals, highly cited authors, milestone publications, and clinical trial development; (3) visualize the evolution of major research clusters; and (4) explore the current research landscape, challenges, and future directions.

## 2 Materials and methods

### 2.1 Data source

Literature was retrieved from the Science Citation Index Expanded (SCI-Expanded) of the WoSCC and from the PubMed database for the period from 2000 to 2025. The search strategies for both databases were developed with reference to seven key studies (14–20). For WoSCC, the core search structure was: TS = (“COPD” OR “Chronic Obstructive Pulmonary Disease” OR related COPD synonyms) AND TS = (“mHealth” OR “mobile health” OR “telehealth” OR “telemedicine” OR related mobile or portable digital health terms). For PubMed, the search similarly combined COPD-related terms, including “Pulmonary Disease, Chronic Obstructive” [MeSH], with mHealth-related terms, including “Telemedicine” [MeSH], and was restricted to English-language clinical trials or randomized controlled trials. The complete search strategies, including all synonyms, Boolean operators, database-specific syntax, document type restrictions, language limits, and timespan filters, are provided in Supplementary Table 1 for WoSCC and Supplementary Table 2 for PubMed. A parallel dual-database design was adopted because the two sources served complementary purposes: WoSCC provides structured citation metadata, including cited references and citation links, which are suitable for bibliometric network analyses, whereas PubMed was used to capture clinically indexed trial evidence and translational developments. This design enabled the study to combine macro-level mapping of the research landscape with focused analysis of clinical trial progress. It also avoided the structural heterogeneity that may arise from directly merging the two databases. Scopus and Embase were not additionally included because directly merging records from multiple bibliographic databases might cause metadata heterogeneity, duplicate overlap, and inconsistencies in citation fields, which could affect the reliability and comparability of network analyses.

### 2.2 Literature screening inclusion and exclusion criteria

**Figure 2:**
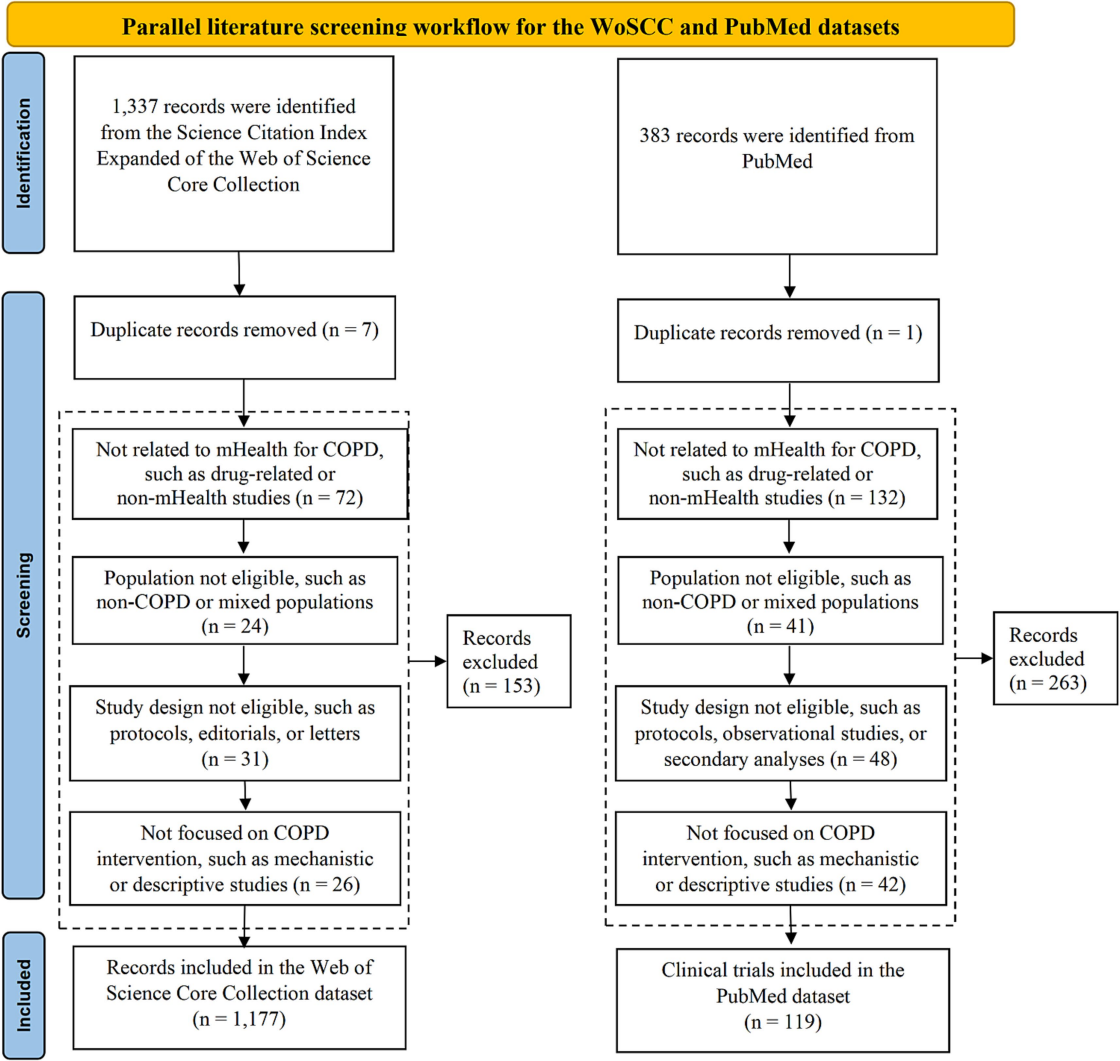
Parallel literature screening workflow for the WoSCC dataset and the PubMed dataset.

Literature screening was performed to select studies for this review. The screening process followed the BIBLIO checklist (Supplementary Table 3), a reporting guideline for biomedical bibliometric reviews, to ensure methodological transparency and rigor. Two independent reviewers screened the records, and any discrepancies were resolved through discussion or, when necessary, consultation with a third senior researcher. Because the WoSCC and PubMed datasets served different analytical purposes, their screening workflows were conducted in parallel, as shown in Figure 2.

For the WoSCC dataset, which was used for macro-level bibliometric analysis, the initial search identified 1,337 records. Database-level filters were first applied to retain English-language original articles and reviews, after which the titles and abstracts were manually screened for relevance. Finally, 1,177 eligible records were included for bibliometric network analyses using CiteSpace and VOSviewer.

For the PubMed dataset, which was used for clinical trial analysis, the initial search identified 383 records. To specifically capture clinical evidence, database-level filters were first applied to retain English-language clinical trials and randomized controlled trials, followed by manual screening for relevance. Finally, 119 clinical trials were included for analysis of translational trends in mHealth for COPD.

Because the two datasets served different analytical purposes and differed in data structure and export formats, they were processed in parallel rather than merged. This approach helped preserve data integrity, maintain analytical consistency within each dataset, and avoid format incompatibility in subsequent visualization analyses. Duplicate checking was performed within each database export before screening (21).

### 2.3 Visualized and bibliometric analysis

CiteSpace is a scientific literature analysis software developed for visualizing and analyzing academic data (21). In this study, CiteSpace was applied to the WoSCC and PubMed datasets to perform keyword clustering, citation burst detection, and timeline visualization, thereby characterizing research hotspots, thematic structures, and temporal evolution. The time span was set from 2000 to 2025 with 1 year per slice, and node selection was based on the g-index (*k* = 25). For keyword clustering and timeline analyses, keywords were selected as the node type, cluster labels were extracted using the log-likelihood ratio (LLR) algorithm, and cosine similarity was used to measure link strength within slices. No pruning algorithm was applied, and this decision was made to preserve the complete and original topological structure of the networks. Citation burst detection was performed using Kleinberg’s algorithm, with figure-specific parameters, including *γ* values, minimum burst duration, and displayed burst items, provided in the corresponding figure legends. For the PubMed clinical trial dataset, although the retrieval period covered 2000–2025, the visualized timeline began in 2008 because earlier low-frequency terms were filtered out under the node selection threshold.

VOSviewer (version 1.6.20) was used to construct network maps of co-authorship, co-occurrence, citation, and co-citation relationships (22). The association strength method was used to normalize link strengths in the VOSviewer networks, and analysis-specific thresholds for publication output, citation frequency, co-citation frequency, or keyword occurrence frequency were set according to the characteristics of each network; detailed thresholds and visualization parameters are provided in the corresponding figure legends. ArcMap was used to visualize the global distribution of publications, and Microsoft Excel 365 was used for descriptive statistical analysis. HistCite Pro 2.1 was used to calculate Local Citation Scores (LCS) and Global Citation Scores (GCS), which were then used to identify influential publications within and beyond the included dataset (23). In HistCite Pro 2.1, the “limit” parameter was set to 30, while all other settings were kept at their default values (24).

## 3 Results

### 3.1 Publication output trend

The WoSCC dataset finally included 1,177 publications (932 research articles and 245 review articles), involving 6,159 co-authors and 2,275 institutions from 83 countries. These papers were published in 319 journals and collectively cited 35,263 references from 10,392 distinct journals.

**Figure 3:**
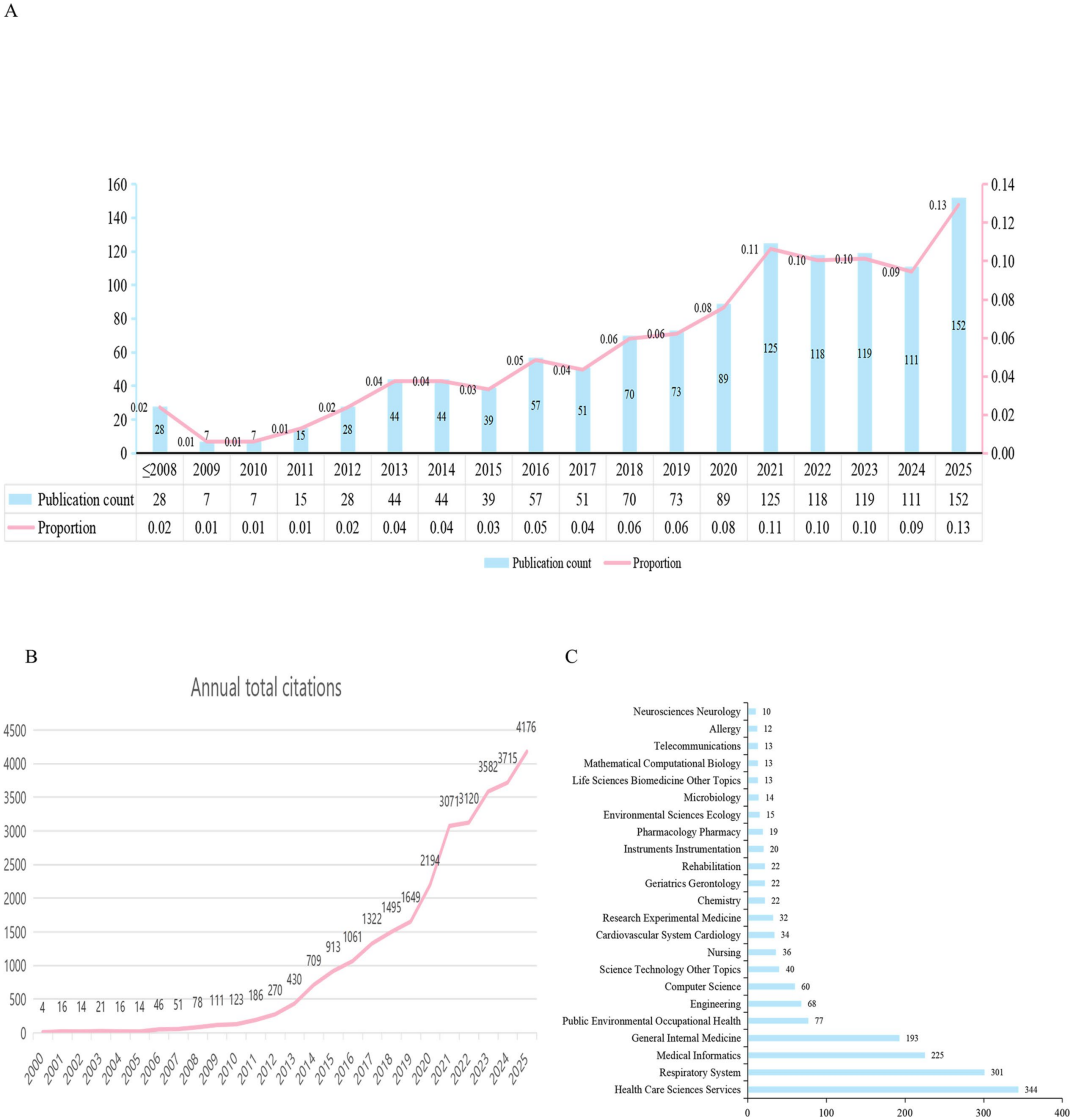
**(A)** Annual publication output and corresponding proportion of publications from 2000 to 2025 based on the WoSCC dataset. The bar chart represents the number of publications per year, while the line chart indicates the proportion of annual publications relative to the total number of included WoSCC publications (*n* = 1,177). **(B)** Annual total citations received by the included WoSCC publications in the field. **(C)** Distribution of publications by research discipline in mHealth research for COPD.

As depicted in Figure 3A, annual publication output showed an overall upward trend with some fluctuations over time, increasing from 28 papers in or before 2008 to 152 papers in 2025. The growth trajectory could be broadly divided into three phases: an early exploratory stage (≤2012), a period of steady growth (2013–2019), and a phase of accelerated expansion (2020–2025). The corresponding annual proportion, calculated as the annual publication count divided by the total number of included WoSCC publications (*n* = 1,177), also rose from 0.02 to 0.13, reflecting the increasing prominence of this research field. Figure 3B further reflected the rising academic impact of this field, as the total annual citations received by the included WoSCC publications increased markedly from 4 in 2000 to 4,176 in 2025, indicating growing recognition and influence of relevant studies. Meanwhile, Figure 3C revealed the interdisciplinary nature of this research, with the highest publication volumes concentrated in Health Care Sciences Services (344), Respiratory System (301), and Medical Informatics (225), indicating that this field developed at the intersection of respiratory medicine, health services, and medical informatics.

### 3.2 Analysis of journals and cited journals

#### 3.2.1 Analysis of journals

Identifying leading journals within a research field assists scholars in aligning their work with key publication outlets, thereby facilitating the recognition of predominant research themes and trajectories.

**Figure 4:**
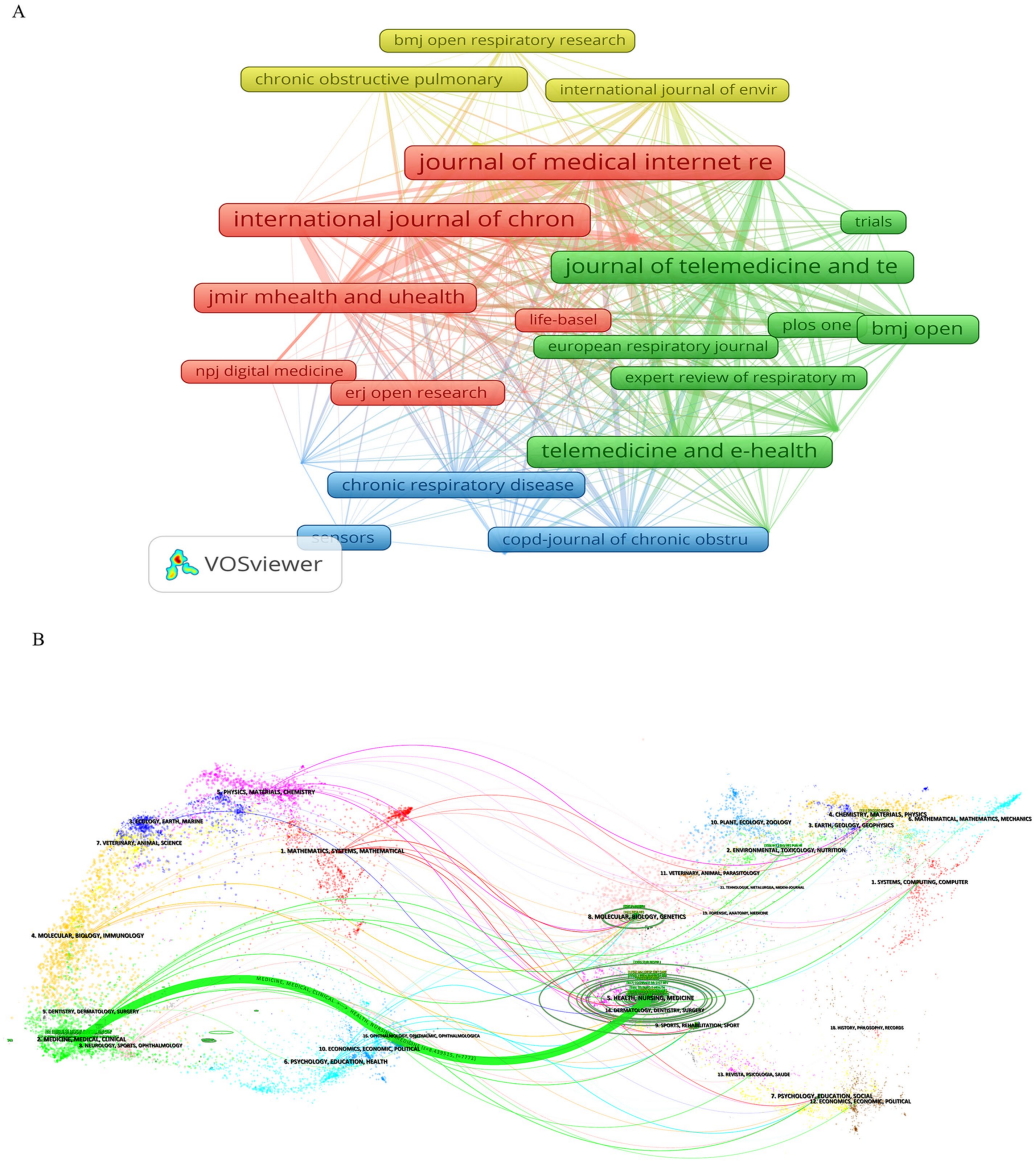
Journal citation network and dual-map overlay of mHealth research for COPD. **(A)** Journal citation network generated using VOSviewer. The minimum threshold for inclusion was set at 9 publications per journal. Node size represents citation frequency, line thickness indicates link strength between journals, and colors denote journal clusters. **(B)** Dual-map overlay showing citation trajectories between citing journals on the left and cited journals on the right.

To characterize the major publication venues in this field, the top journals were ranked according to publication output. Table 1 presents the top 10 leading journals in mHealth research for COPD. The *Journal of Medical Internet Research* (*JMIR*) ranked first in publication volume, followed by the *International Journal of Chronic Obstructive Pulmonary Disease* (*IJCOPD*). In terms of citation performance, the *Journal of Telemedicine and Telecare* had the highest total citations (2,091), whereas *BMC Health Services Research* showed the highest average citations per publication (48.94). By contrast, the *JMIR mHealth and uHealth* had the highest journal impact factor (6.20) among the top journals listed in Table 1. The citation network further revealed close citation links among several leading journals, including *JMIR*, *IJCOPD*, and *JMIR mHealth and uHealth* (Figure 4A). This pattern indicated that the mHealth and COPD clinical research domains were actively informing each other, delineating a cohesive research community. In addition, the journal dual-map overlay showed that the dominant citation trajectory in this field ran from *medicine/medical/clinical* journals to *health/nursing/medicine* journals (Figure 4B), suggesting that the knowledge base of this field was centered primarily within the health and medical sciences. Together, these findings suggest that mHealth research in COPD was grounded primarily in a clinically oriented knowledge base.

**Table 1:** Top 10 journals by publication output in mHealth research for COPD.

Journals	Documents	Citations	Average citations	Journal citation reports quartile	Journal impact factor
*Journal of Medical Internet Research*	65	1898	29.20	Q1	6.00
*International Journal of Chronic Obstructive Pulmonary Disease*	60	978	16.30	Q2	3.10
*Journal of Telemedicine and Telecare*	50	2091	41.82	Q2	3.20
*Telemedicine and e-Health*	45	1,381	30.69	Q3	2.00
*JMIR mHealth and uHealth*	36	1,120	31.11	Q1	6.20
*BMJ Open*	34	464	13.65	Q2	2.30
*Respiratory Medicine*	20	599	29.95	Q2	3.10
*BMC Medical Informatics and Decision Making*	19	548	28.84	Q2	3.80
*BMC Health Services Research*	18	881	48.94	Q2	3.00
*Chronic Respiratory Disease*	18	535	29.72	Q2	2.30

#### 3.2.2 Analysis of co-cited journals

**Figure 5:**
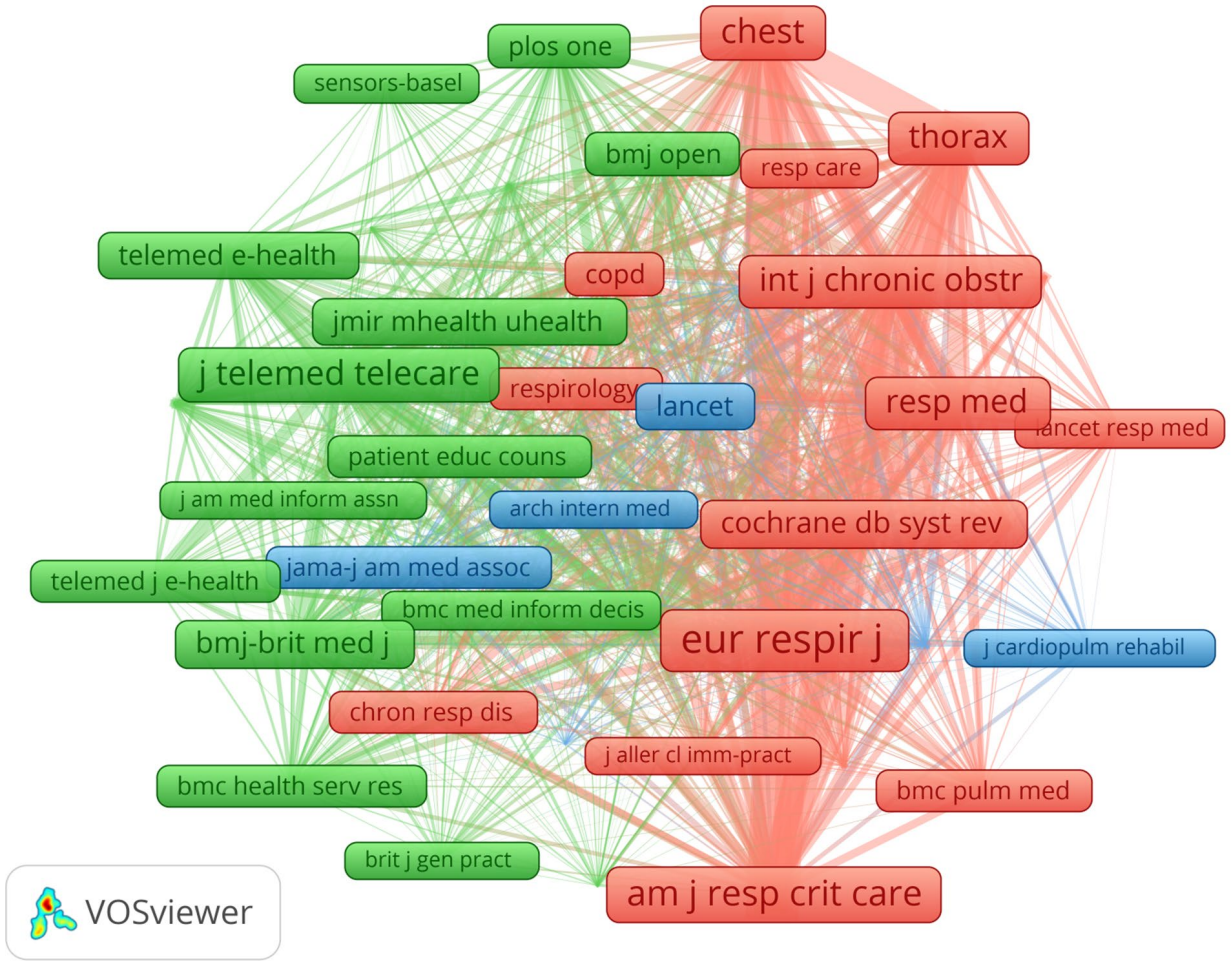
Co-cited journal network of mHealth research in COPD. The co-citation map was generated using VOSviewer. The minimum threshold for inclusion was set at 150 co-citations per journal. Node size represents co-citation frequency, line thickness indicates co-citation link strength between journals, and colors denote journal clusters.

Analyzing journal co-citations helps identify interrelated academic fields and visualize the network of scholarly communication, thereby informing systematic literature reviews. In the findings, the leading co-cited journals were listed in Table 2, and a graphical representation of their relationships was provided in Figure 5.

**Table 2:** Top 10 co-cited journals in mHealth research for COPD.

Journals	Co-citations	Journal citation reports quartile	Journal impact factor
*European Respiratory Journal*	1994	Q1	21.20
*American Journal of Respiratory and Critical Care Medicine*	1,463	Q1	19.40
*Journal of Medical Internet Research*	1,303	Q1	6.00
*CHEST*	1,220	Q1	9.20
*Journal of Telemedicine and Telecare*	1,219	Q2	3.20
*International Journal of Chronic Obstructive Pulmonary Disease*	1,174	Q2	3.10
*Thorax*	1,130	Q1	8.30
*Respiratory Medicine*	1,086	Q2	3.10
*Cochrane Database of Systematic Reviews*	848	Q1	9.40
*BMJ-British Medical Journal*	745	Q1	43.00

Among the most frequently co-cited journals, the *European Respiratory Journal* ranked first, underscoring the foundational role of core respiratory medicine research in this domain. The second most co-cited journal, the *American Journal of Respiratory and Critical Care Medicine*, further emphasized the centrality of rigorous clinical research in respiratory and critical care medicine. Notably, the *Journal of Medical Internet Research* ranked third, which clearly highlighted the significant integration and scholarly recognition of mHealth and telemedicine themes within the field.

Figure 5 further showed a clustered co-citation structure, with core respiratory journals (e.g., *European Respiratory Journal*, *American Journal of Respiratory and Critical Care Medicine*, and *Thorax*) forming the main knowledge base, while telemedicine and mHealth journals (e.g., *JMIR mHealth and uHealth*, and *Journal of Telemedicine and Telecare*) occupied a closely connected adjacent cluster, reflecting the interdisciplinary integration of respiratory medicine and digital health research.

### 3.3 Analysis of countries and institutions

#### 3.3.1 Analysis of countries

**Figure 6:**
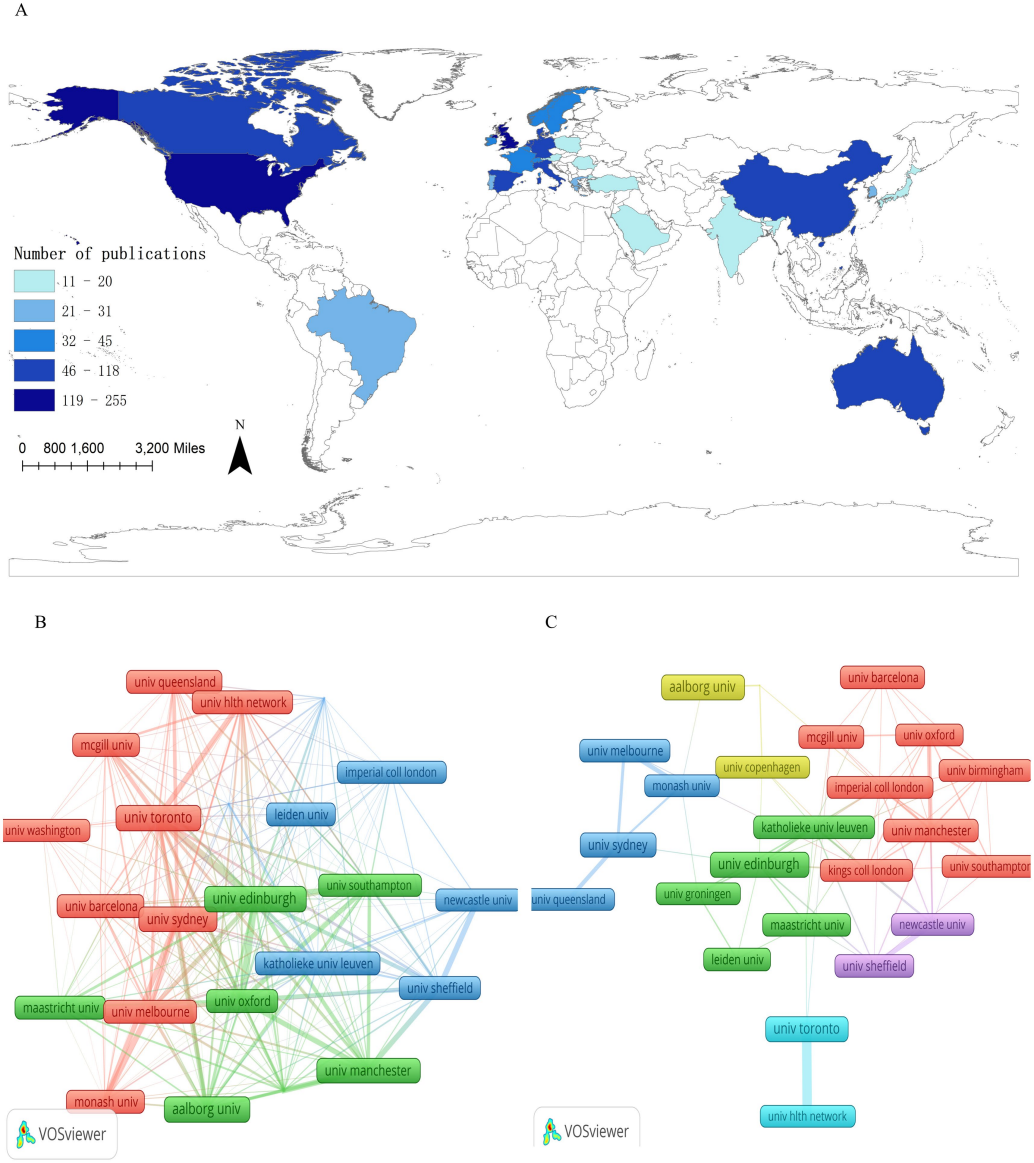
Geographic distribution, institutional citation network, and institutional co-authorship network of mHealth research for COPD. **(A)** Geographic distribution of publications by country. **(B)** Institutional citation network generated using VOSviewer. The minimum threshold for inclusion was set at 14 publications per institution. **(C)** Institutional co-authorship network generated using VOSviewer. The minimum threshold for inclusion was set at 14 publications per institution. **(B,C)** Node size represents publication output, line thickness indicates link strength, and colors denote institutional clusters.

The analysis of national contributions was complemented by the geographical distribution of research productivity illustrated in Figure 6A. As shown in Table 3, the United States led in publication output (254 documents), establishing itself as the most productive country, a finding also visually underscored by the map. England ranked first in total citations, with 6,357 citations from 207 documents. Average citations were calculated as total citations divided by the number of documents for each country. On this basis, Canada, while ranking fifth in publication volume (97 documents), showed the highest average citations (32.09), indicating the strong relative impact of its research. These metrics delineated a collaborative yet competitive international landscape, where the United States dominated in publication output, England ranked first in total citations, and Canada showed the highest average citations.

**Table 3:** Top 10 countries by publication output in mHealth research for COPD.

Countries	Documents	Citations	Average citations
United States	254	5,697	22.43
England	207	6,357	30.71
Netherlands	113	3,346	29.61
China	105	1881	17.91
Canada	97	3,113	32.09
Australia	94	2,729	29.03
Spain	94	2,365	25.16
Italy	83	2,275	27.41
Denmark	80	1966	24.58
Germany	79	1,255	15.89

#### 3.3.2 Analysis of institutions

This study analyzed the top 10 institutions by publication output to identify leading research contributors and delineate their collaborative networks (Table 4).

**Table 4:** Top 10 institutions by publication output in mHealth research for COPD.

Institutions	Documents	Citations	Average citations
University of Toronto	32	848	26.50
Aalborg University	28	601	21.46
University of Edinburgh	28	1,361	48.61
University of Sydney	23	790	34.35
University of Manchester	21	1,375	65.48
University of Southern Denmark	20	429	21.45
University of Sheffield	18	854	47.44
University of Melbourne	18	769	42.72
Maastricht University	18	407	22.61
Leiden University	18	215	11.94

The University of Toronto had the highest number of publications, with 32 publications, which accounted for 2.72% of the total included publications. The University of Manchester had the highest average citation count, with 65.48 citations per publication. Among the top 10 institutions by publication output, all were located in high-income countries across North America, Europe, and Australia. This concentration highlights the leading role of institutions in developed regions in mHealth research for COPD. However, according to the World Health Organization (WHO), nearly 90% of COPD deaths among people under 70 years of age occur in low- and middle-income countries (25). This contrast suggests a geographical imbalance between where research is produced and where the greatest premature mortality burden lies.

This pattern also suggests that the need for effective and accessible mHealth-based management strategies for COPD in low- and middle-income countries has not been adequately addressed by current research paradigms. Therefore, further studies are needed to develop and evaluate context-appropriate solutions in these high-burden and underserved regions.

As illustrated in Figure 6B, the distribution of productive institutions exhibited a pronounced top-tier effect, with most publications and dense collaborative ties concentrated among leading universities in Australia, North America, and Western Europe, such as the University of Queensland, the University of Toronto, and the University of Edinburgh. The institutional co-authorship network further underscored this centrality (Figure 6C): the strongest collaborative link existed between the University of Toronto and its clinical affiliate, the University Health Network. This indicated a tight research alliance, with frequent co-publications between the university’s academic research and the hospital network’s clinical studies. In contrast, institutions outside these core groups occupied marginal positions in the collaborative network, with fewer connections and lower output.

### 3.4 Analysis of authors

**Figure 7:**
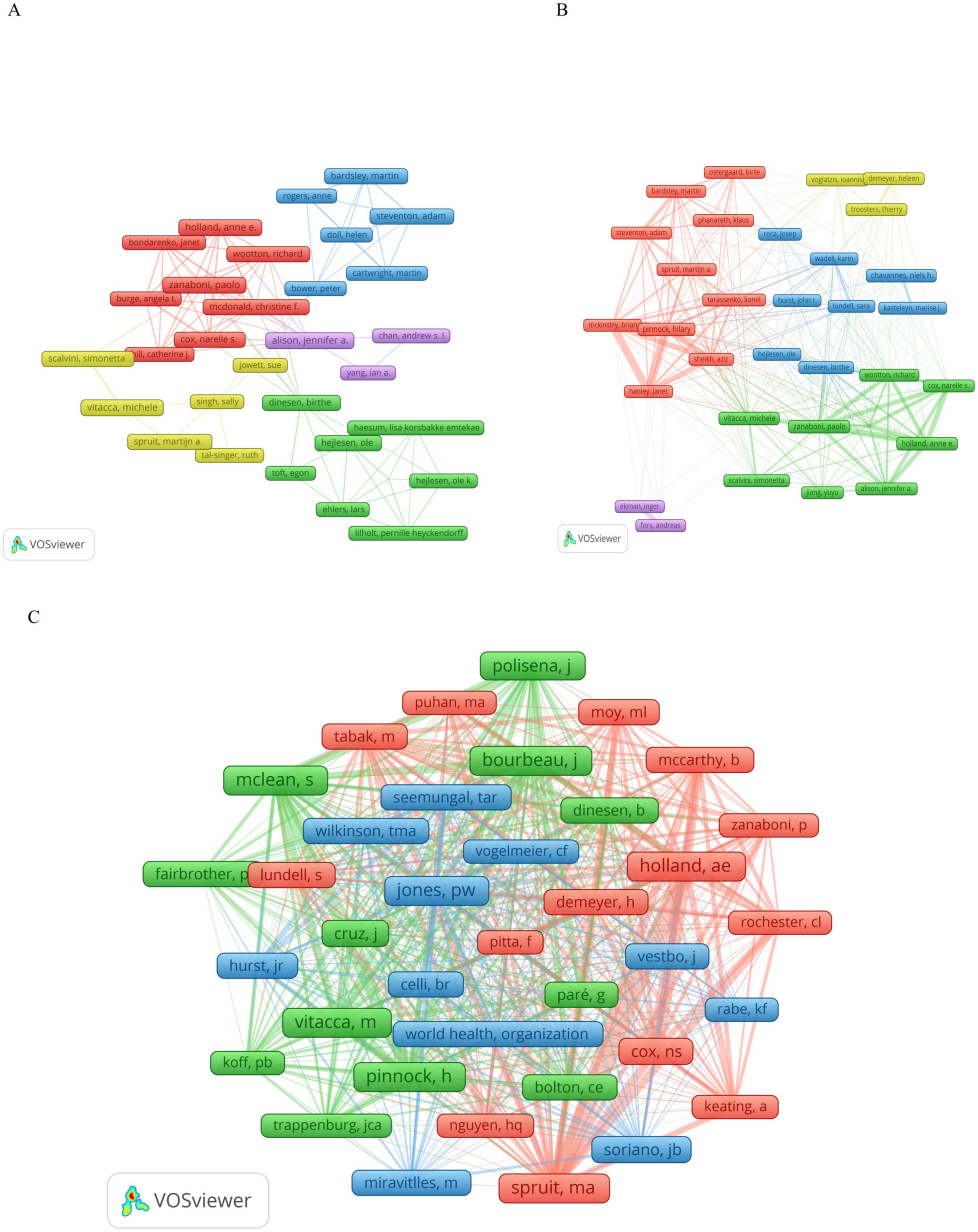
Author co-authorship, citation, and co-citation networks of mHealth research for COPD. **(A)** Author co-authorship network generated using VOSviewer with a threshold of at least 5 publications per author. **(B)** Author citation network generated using VOSviewer with a threshold of at least 7 publications per author. **(C)** Author co-citation network generated using VOSviewer with a threshold of at least 59 citations per author. Node size represents publication output in panel A and citation frequency in **(B,C)**; line thickness indicates link strength, and colors denote author clusters.

Analysis of leading authors helped to identify the major contributors to this field and clarify their collaborative relationships and academic influence. Anne E. Holland and Michele Vitacca ranked first in publication volume, with 13 documents each, whereas Paolo Zanaboni led in both total citations (890) and average citations per publication (80.91). These quantitative findings were further supported by the author network maps (Figure 7). Anne E. Holland was not only one of the most productive authors but also occupied a prominent position in the co-authorship network, where she was closely connected with Paolo Zanaboni, Richard Wootton, Narelle S. Cox, et al., forming an influential collaborative cluster (Figure 7A).

The author citation network further revealed two prominent groups of influential contributors (Figure 7B). One dense cluster was formed by Brian McKinstry, Hilary Pinnock, Janet Hanley, Aziz Sheikh, et al., indicating their close citation relationships and shared academic influence in this field. Another prominent cluster included Richard Wootton, Paolo Zanaboni, Narelle S. Cox, Anne E. Holland, Jennifer A. Alison, et al. In the author co-citation network, authors such as Spruit, Bourbeau, Jones, et al., together with the World Health Organization, occupied prominent positions, indicating their important roles in the knowledge structure of this field (Figure 7C). Overall, these patterns suggested that mHealth research for COPD was shaped not only by highly productive individual authors, but also by several interconnected groups of influential researchers (Table 5).

**Table 5:** Top 10 authors by publication output in mHealth research for COPD.

Authors	Documents	Citations	Average citations
Anne E. Holland	13	556	42.77
Michele Vitacca	13	346	26.62
Paolo Zanaboni	11	890	80.91
Jennifer A. Alison	10	471	47.10
Christine F. McDonald	9	598	66.44
Birthe Dinesen	9	325	36.11
Ole Hejlesen	9	240	26.67
Narelle S. Cox	8	456	57.00
Simonetta Scalvini	8	235	29.38
Martijn A. Spruit	7	201	28.71

### 3.5 Analysis of keywords

#### 3.5.1 Keyword selection and ranking

The analysis of keywords in bibliometrics served to map the field’s evolution, pinpoint active research fronts, and anticipate future directions. High-frequency keywords provided a foundational basis for this analysis by revealing central literature themes.

**Figure 8:**
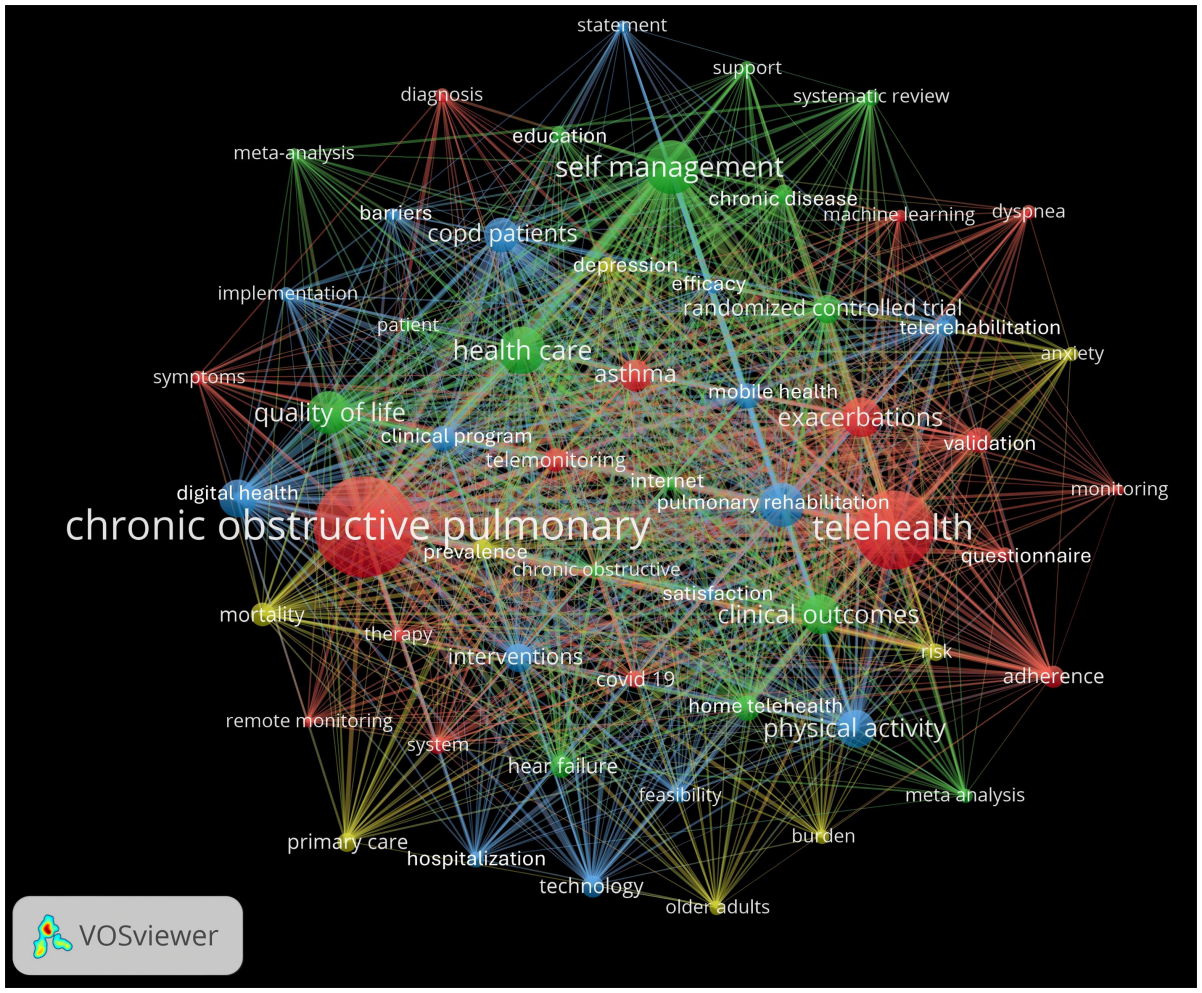
Keyword co-occurrence network of mHealth research for COPD. The network was generated using VOSviewer. Keywords were included if they occurred at least 22 times. Node size represents keyword occurrence frequency, line thickness indicates co-occurrence link strength, and colors denote keyword clusters.

According to Price’s Law (26), the minimum frequency (*m*) for a keyword to be considered high-frequency was calculated as: m = 0.749 × $\sqrt{N\max},$ where *N*_max_ represents the occurrence frequency of the most frequent keyword. In this study, the highest-frequency keyword appeared 857 times, yielding a minimum threshold of approximately 21.93. Accordingly, the minimum frequency for inclusion as a high-frequency keyword was set at 22. Based on this criterion, a keyword co-occurrence map was generated (Figure 8).

The top 10 high-frequency keywords were presented in Table 6. The four most frequently occurring keywords were: chronic obstructive pulmonary disease (857), telehealth (558), self management (293), and health care (236).

**Table 6:** Top 10 keywords by occurrence frequency in mHealth research for COPD.

Keywords	Occurrences	Total link strength
Chronic obstructive pulmonary disease	857	3,487
Telehealth	558	2,485
Self management	293	1,506
Health care	236	1,153
Pulmonary rehabilitation	207	1,063
Quality of life	192	981
Exacerbations	175	892
Clinical outcomes	168	875
Physical activity	157	747
Digital health	155	721

These findings indicated that current research hotspots primarily centered on chronic obstructive pulmonary disease itself, with telehealth and digital health representing the main technological modalities, self-management and pulmonary rehabilitation representing core intervention pathways, and quality of life, exacerbations, physical activity, and clinical outcomes serving as major outcome-related themes. Together, these keywords suggested a closely connected research framework focused on technology-enabled, patient-centered COPD management.

#### 3.5.2 Keyword co-occurrence clustering analysis

**Figure 9:**
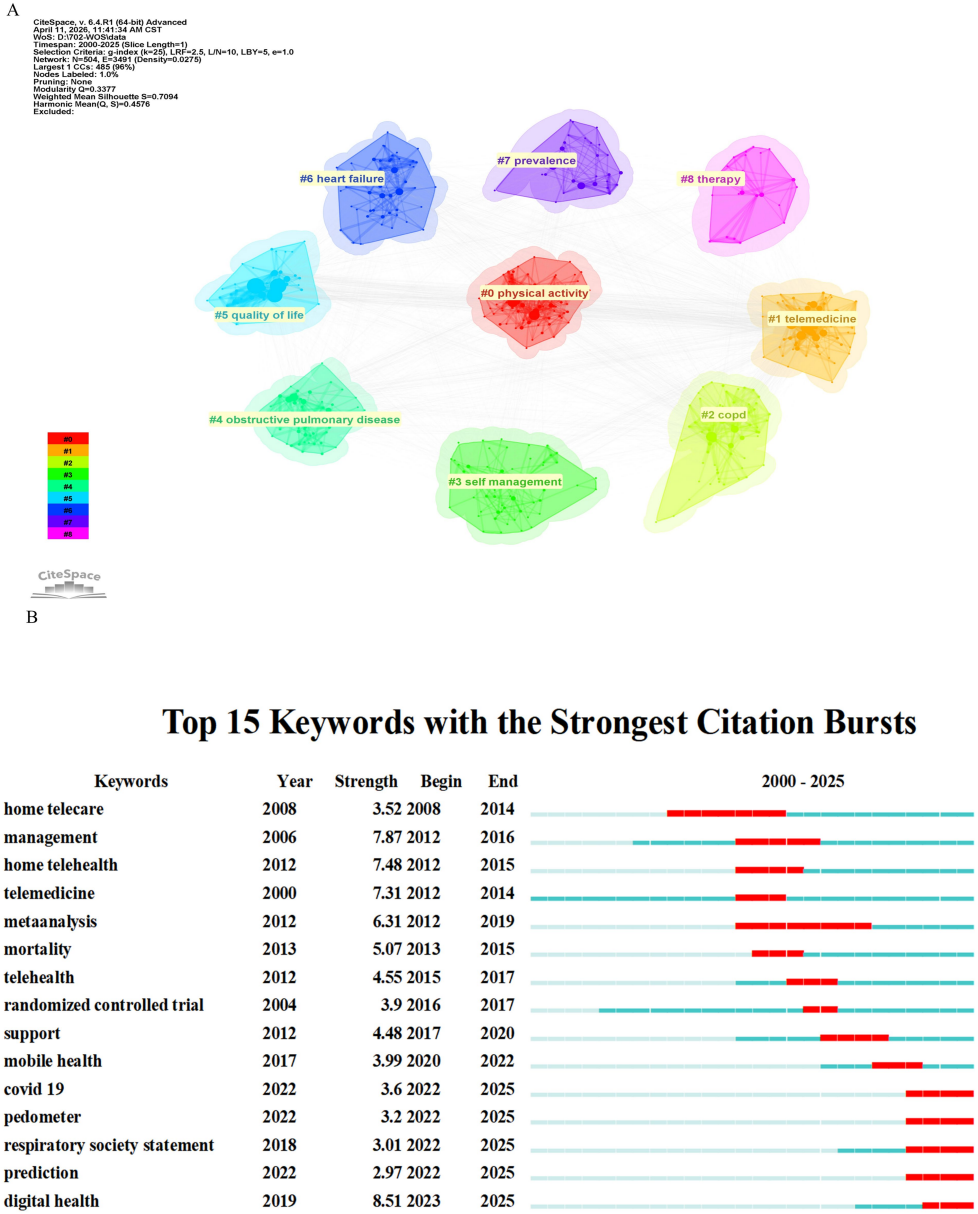
Keyword clustering and burst analysis in mHealth research for COPD. CiteSpace was used for keyword clustering and burst detection, with the time span set from 2000 to 2025 and 1 year per slice. **(A)** Keyword co-occurrence clustering map. Keywords were selected as the node type, node selection was based on the *g*-index (*k* = 25), cluster labels were extracted using the log-likelihood ratio algorithm, and no pruning was applied. **(B)** Keyword burst analysis. Burst detection was performed using Kleinberg’s algorithm with 2 states, *γ* = 0.6, and a minimum duration of 2 years; the top 15 keywords with the strongest burst intensity are displayed.

Cluster analysis divided the research keywords into eight distinct thematic clusters (Figure 9A). #2 and #4 clusters directly addressed the disease itself. The remaining six clusters covered multiple research dimensions in this field. Notably, “telemedicine” formed a clearly independent cluster. This finding suggests that telemedicine has evolved from a secondary topic under the general disease theme into an important research focus in this field. Collectively, these clusters outlined the comprehensive research scope of the domain, expanding from the fundamental research on the disease entity to the exploration of diverse intervention strategies, clinical management approaches and prognostic evaluation indicators for the disease.

#### 3.5.3 Keyword burst analysis

Keyword burst analysis helped identify temporal shifts in research focus and highlighted emerging areas of interest. As shown in Figure 9B, the term ‘digital health’ exhibited the strongest burst intensity (8.51), which reflected its rising dominance as a core research direction in the field. Recent burst keywords included “covid 19,” “pedometer,” and “respiratory society statement,” all of which were expected to remain influential. These terms reflected the growing integration of digital health technologies into respiratory disease management. This trend was accelerated by the COVID-19 pandemic and accompanied by increasing attention to objective activity monitoring and evidence-based clinical guidance.

An analysis of keyword bursts also revealed a phased evolution in the field: (1) Early stage (2008–2014): Focused on the initial exploration of telehealth modalities, with keywords such as “home telecare” and “telemedicine” indicating foundational research into remote care delivery models for respiratory diseases. (2) Mid-stage (2015–2020): This stage was marked by the clinical maturation and methodological refinement of telehealth applications. Keywords such as “telehealth,” “randomized controlled trial,” and “support” reflected efforts to validate telehealth efficacy and explore supportive care strategies. The emergence of “mobile health” also indicated an initial shift toward portable digital interventions. (3) Recent stage (2022–2025): This stage reflected digital transformation and pandemic-driven priority shifts. Keywords such as “digital health,” “covid 19,” and “pedometer” indicated the integration of digital health frameworks into clinical practice, the impact of the COVID-19 pandemic, and the growing use of wearable devices for objective activity assessment. This progression suggested that the field had evolved from the early exploratory phase of basic telehealth models to a sophisticated landscape of data-driven, technology-enabled respiratory disease management.

### 3.6 Analysis of cited reference

#### 3.6.1 The top 10 cited documents analysis

Analysis of citation patterns served to pinpoint the most influential core publications within the field, thereby guiding subsequent research. The top 10 most cited publications were highlighted in Table 7, which also summarized their main research focuses (27–35). A closer look at the timeline showed that the highly cited literature was concentrated between 2010 and 2014, with 3 papers published in 2012 alone, indicating this was a key period of accumulation for telehealth research. The earliest paper (2000) focused on cost-effectiveness, while the most recent (2021) focused on equivalence to traditional interventions and practical feasibility. This shift reflected the field’s evolution from validating basic effectiveness to optimizing practical application.

**Table 7:** Top 10 cited publications in mHealth research for COPD.

Documents	First author	Publication year	Citations	Main Findings
*Twenty years of telemedicine in chronic disease management-an evidence synthesis*	Richard Wootton (27)	2012	358	This paper primarily examined the value of telemedicine in managing multiple chronic diseases. It found that although most studies reported positive effects, the overall evidence base was weak and contradictory due to potential publication bias, short-term study durations, and a lack of cost-effectiveness analyses.
*Telerehabilitation for chronic respiratory disease*	Narelle S Cox (8)	2021	327	This paper primarily assessed telerehabilitation for chronic respiratory disease. It concluded that telerehabilitation was as effective as traditional rehabilitation with higher completion rates, but the evidence certainty was limited by the small number of studies.
*Exploring barriers to participation and adoption of telehealth and telecare within the Whole System Demonstrator trial: a qualitative study*	Caroline Sanders (28)	2012	315	This paper used qualitative interviews to explore why people declined or withdrew from telehealth interventions. It found key barriers included misunderstandings about required technical competence, perceptions that the intervention threatened positive self-identity and independence, and concerns about disruption to valued existing services.
*A systematic review of web-based interventions for patient empowerment and physical activity in chronic diseases: relevance for cancer survivors*	Wilma Kuijpers (29)	2013	293	This paper systematically reviewed interactive Web-based interventions for patient empowerment and physical activity across chronic diseases. It found mixed effects but identified seven common intervention elements that could inform the design of similar eHealth approaches for cancer survivors.
*Outcomes of the Kaiser Permanente Tele-Home Health Research Project*	B. Johnston (30)	2000	248	This paper evaluated the use of remote video technology in home health care. It found the technology was effective in maintaining quality of care, well-received by patients, and demonstrated the potential to reduce overall care costs.
*Effectiveness of telemonitoring integrated into existing clinical services on hospital admission for exacerbation of chronic obstructive pulmonary disease: researcher blind, multicenter, randomized controlled trial*	Hilary Pinnock (31)	2013	239	This study evaluated integrating telemonitoring into standard COPD care and found it did not significantly improve time to hospital admission or quality of life. Its value lay in critically reframing the technology’s role: telemonitoring might not be a standalone intervention, but a component for enhancing clinical processes.
*The empirical foundations of telemedicine interventions for chronic disease management*	Rashid L. Bashshur (32)	2014	230	This review assessed the impact of telemedicine on managing three chronic diseases: heart failure, stroke, and COPD. It found that the majority of methodologically rigorous studies demonstrated telemonitoring effectively reduced healthcare utilization and mortality, with only a few reporting neutral or mixed findings.
*Effect of telehealth on use of secondary care and mortality: findings from the Whole System Demonstrator cluster randomized trial*	Adam Steventon (33)	2012	226	This randomized controlled trial involving patients with diabetes, COPD, or heart failure found that those receiving home-based telehealth had significantly lower hospital admission rates, mortality, and shorter average hospital stays over 12 months compared to the usual care group.
*Home telehealth for chronic obstructive pulmonary disease: a systematic review and meta-analysis*	Julie Polisena (34)	2010	221	This systematic review on COPD found that home telehealth reduced hospitalizations and emergency visits, and performed similarly or better than usual care in quality of life and satisfaction. However, the telephone-support subgroup showed a trend toward higher mortality.
*Tele-assistance in chronic respiratory failure patients: a randomized clinical trial*	M. Vitacca (35)	2009	200	This study on patients with chronic respiratory failure requiring oxygen or home mechanical ventilation found that a one-year, nurse-centred tele-assistance programme significantly reduced hospitalisations, acute exacerbations, and urgent general practitioner calls, and was cost-effective. The subgroup of COPD patients appeared to derive particular benefit.

#### 3.6.2 Reference bursts analysis

**Figure 10:**
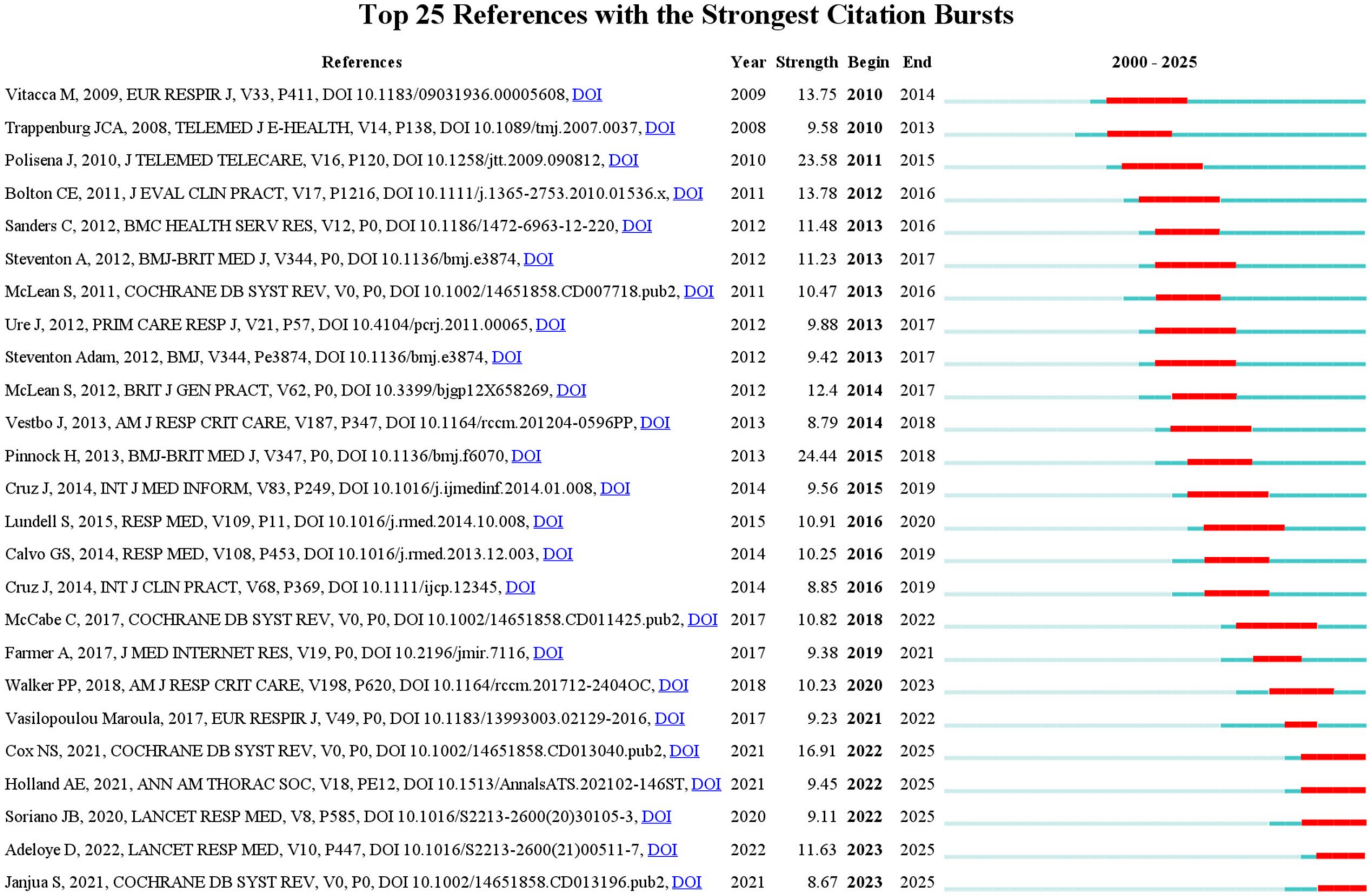
Reference citation bursts in mHealth research for COPD. CiteSpace was used to detect reference citation bursts, with the time span set from 2000 to 2025 and 1 year per slice. Burst detection was performed using Kleinberg’s algorithm with 2 states, γ = 1.0, and a minimum duration of 2 years; the top 25 references with the strongest burst strength are displayed.

Reference bursts could reflect changes in the attention received by references in the field, thereby indicating shifts in research hotspots. As shown in Figure 10, reference bursts active in the past five years included works by Cox NS (2021) (8), Holland AE (2021) (36), Soriano JB (2020) (37), Adeloye D (2022) (15), and Janjua S (2021) (7). These highly cited publications collectively delineated COPD as a critical global health challenge. These studies evaluated telemedicine and rehabilitation as emerging COPD management strategies. They suggested potential benefits in access, selected clinical outcomes, and cost-effectiveness, although the overall strength of evidence and optimal implementation models still require further clarification.

#### 3.6.3 Reference impact analysis

HistCite was used to evaluate the citation impact of publications within and beyond the included dataset. Local Citation Score (LCS) refers to the number of times a publication was cited by other publications within the local collection, whereas Global Citation Score (GCS) refers to the citation frequency based on the full Web of Science count at the time the data were downloaded (23, 24). In this study, the local collection referred to the included WoSCC dataset. Therefore, LCS was used to reflect the field-specific influence of a publication within mHealth research for COPD, while GCS was used to indicate its broader academic impact.

The HistCite analysis identified the 30 publications with the highest LCS within the dataset (34, 35, 38–64) (Table 8). The systematic review by Polisena et al. (2010) (34) had the highest LCS (101), indicating that it was the most frequently cited publication within the included WoSCC dataset. In contrast, the evidence synthesis by Wootton (2012) (27) had the highest GCS (358) but a lower LCS (32), suggesting that although this publication had broad citation impact across the wider field of telemedicine and chronic disease management, its citation impact within the more specific field of mHealth research for COPD was relatively lower.

**Table 8:** Top 30 publications ranked by Local Citation Score in mHealth research for COPD.

NO.	Article information	Journal	LCS	GCS	First author
1	*Home telehealth for chronic obstructive pulmonary disease: a systematic review and meta-analysis*	*Journal of Telemedicine and Telecare*	101	221	Julie Polisena (34)
2	*Tele-assistance in chronic respiratory failure patients: a randomized clinical trial*	*European Respiratory Journal*	70	200	M Vitacca (35)
3	*Telehealthcare in COPD: A systematic review and meta-analysis on physical outcomes and dyspnea*	*Respiratory Medicine*	58	138	Sara Lundell (38)
4	*Insufficient evidence of benefit: a systematic review of home telemonitoring for COPD*	*Journal of Evaluation in Clinical Practice*	53	98	Charlotte E Bolton (39)
5	*Proactive integrated care improves quality of life in patients with COPD*	*European Respiratory Journal*	52	163	P B Koff (40)
6	*Home telemonitoring in COPD: A systematic review of methodologies and patients’ adherence*	*International Journal of Medical Informatics*	52	114	Joana Cruz (41)
7	*Piloting tele-monitoring in COPD: a mixed methods exploration of issues in design and implementation*	*Primary Care Respiratory Journal*	49	89	Jenny Ure (42)
8	*A telehealth program for self-management of COPD exacerbations and promotion of an active lifestyle: a pilot randomized controlled trial*	*International Journal of Chronic Obstructive Pulmonary Disease*	49	131	Monique Tabak (43)
9	*A home telehealth program for patients with severe COPD: the PROMETE study*	*Respiratory Medicine*	49	100	G Segrelles Calvo (44)
10	*Using Telehealth technology to deliver pulmonary rehabilitation to patients with chronic obstructive pulmonary disease*	*Canadian Respiratory Journal*	45	118	Michael Stickland (45)
11	*Home telemonitoring effectiveness in COPD: a systematic review*	*International Journal of Clinical Practice*	45	102	J Cruz (46)
12	*Using preventive home monitoring to reduce hospital admission rates and reduce costs: a case study of telehealth among chronic obstructive pulmonary disease patients*	*Journal of Telemedicine and Telecare*	44	80	Birthe Dinesen (47)
13	*A randomized clinical trial of the effectiveness of home-based health care with telemonitoring in patients with COPD*	*Journal of Telemedicine and Telecare*	43	69	Janet E McDowell (48)
14	*A feasibility study to investigate the acceptability and potential effectiveness of a telecare service for older people with chronic obstructive pulmonary disease*	*International Journal of Medical Informatics*	42	90	Janita Pak-Chun Chau (49)
15	*Physical activity is increased by a 12-week semiautomated telecoaching programme in patients with COPD: a multicentre randomised controlled trial*	*Thorax*	41	191	H Demeyer (50)
16	*Pilot Study of Remote Telemonitoring in COPD*	*Telemedicine and e-Health*	40	62	Nick C Antoniades (51)
17	*Using a mobile health application to support self-management in COPD: a qualitative study*	*British Journal of General Practice*	39	93	Veronika Williams (52)
18	*Telemonitoring in Chronic Obstructive Pulmonary Disease (CHROMED) A Randomized Clinical Trial*	*American Journal of Respiratory and Critical Care Medicine*	39	112	Paul P Walker (53)
19	*Effect of tele health care on exacerbations and hospital admissions in patients with chronic obstructive pulmonary disease: a randomized clinical trial*	*International Journal of Chronic Obstructive Pulmonary Disease*	36	61	Thomas Ringbæk (54)
20	*Continuity, but at what cost? The impact of telemonitoring COPD on continuities of care: a qualitative study*	*Primary Care Respiratory Journal*	35	60	Peter Fairbrother (55)
21	*Twenty years of telemedicine in chronic disease management - an evidence synthesis*	*Journal of Telemedicine and Telecare*	32	358	Richard Wootton (27)
22	*Telemedicine in COPD: An Overview by Topics*	*COPD: Journal of Chronic Obstructive Pulmonary Disease*	30	66	Miguel T Barbosa (56)
23	*Nurse tele-consultations with discharged COPD patients reduce early readmissions - an interventional study*	*Clinical Respiratory Journal*	28	59	Anne Dichmann Sorknaes (57)
24	*Randomised crossover trial of telemonitoring in chronic respiratory patients (TeleCRAFT trial)*	*Thorax*	28	84	M Chatwin (58)
25	*The effect of real-time teleconsultations between hospital-based nurses and patients with severe COPD discharged after an exacerbation*	*Journal of Telemedicine and Telecare*	27	58	Anne Dichmann Sorknaes (59)
26	*Home telemonitoring and quality of life in stable, optimised chronic obstructive pulmonary disease*	*Journal of Telemedicine and Telecare*	26	47	Keir E Lewis (60)
27	*Exploring telemonitoring and self-management by patients with chronic obstructive pulmonary disease: A qualitative study embedded in a randomized controlled trial*	*Patient Education and Counseling*	26	75	Peter Fairbrother (61)
28	*Home telecare for COPD/CHF patients: outcomes and perceptions*	*Journal of Telemedicine and Telecare*	25	68	Pamela Whitten (62)
29	*Efficacy of a cell phone-based exercise program for COPD*	*European Respiratory Journal*	24	126	W-T Liu (63)
30	*Telemedicine in COPD: Time to Pause*	*CHEST*	24	46	Roger S Goldstein (64)

### 3.7 Analysis of clinical trial progress

**Figure 11:**
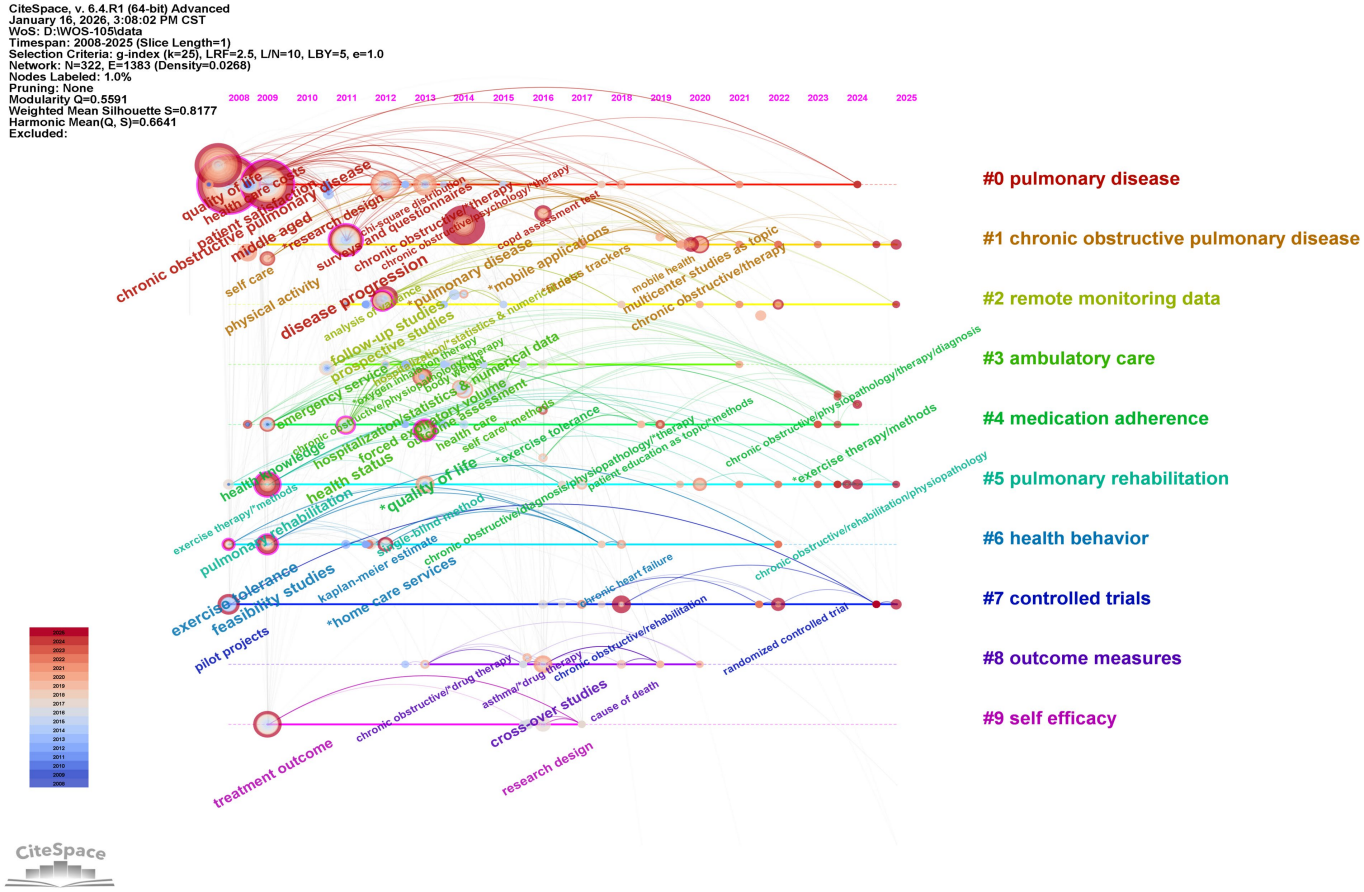
Timeline clustering map of clinical trial-related keyword co-occurrence in mHealth research for COPD. CiteSpace was used to generate the keyword timeline clustering map, with the time span set from 2008 to 2025 and 1 year per slice. Keywords were selected as the node type, node selection was based on the g-index (*k* = 25), cluster labels were extracted using the log-likelihood ratio algorithm, and no pruning was applied.

This study selected PubMed for its repository of high-quality clinical trials to analyze clinical progress. After screening, 119 PubMed clinical trials were finally included in this part of the analysis. The objective was to map current research priorities and emerging directions. Keywords provided a focused view of the field’s central themes. By generating a timeline clustering map of keyword co-occurrence (Figure 11) and examining high-frequency terms (Table 9), the analysis identified the main topics and dynamics in clinical research.

**Table 9:** Top 10 keywords related to clinical trials by occurrence frequency in mHealth research for COPD.

Keywords	Frequency	Centrality
Chronic obstructive pulmonary disease	103	0.12
Middle aged	64	0.11
Quality of life	52	0.09
Pulmonary disease	41	0.08
Treatment outcome	27	0.08
Disease progression	21	0.16
Surveys and questionnaires	19	0.04
Pulmonary rehabilitation	15	0.11
Time factors	15	0.05
Prospective studies	14	0.10

#### 3.7.1 Methodological foundations and design evolution

The “controlled trials” cluster was located in the lower-right peripheral area of the timeline map and included methodological terms such as “randomized controlled trial” (Figure 11). This distribution suggested that trial design related terms were present but were not the dominant thematic hub of the keyword network. The presence of terms such as “randomized controlled trial,” “prospective studies” and “treatment outcome” indicated that prospective and outcome-oriented designs were commonly used to evaluate digital health interventions for COPD. However, current clinical evidence still had several limitations: numerous trials reporting positive outcomes were constrained by single-center designs, small sample sizes, short follow-up periods, and heterogeneous outcome measures (65, 66). In addition, “surveys and questionnaires” appeared among the top 10 keywords, with a frequency of 19 but a relatively low centrality of 0.04 (Table 9), suggesting that patient-reported assessment tools were frequently used but had not yet become major bridging elements in the clinical trial keyword network. The presence of feasibility studies and pilot projects also reflected an exploratory stage in the development of some interventions. Collectively, these limitations chart a clear path for future research: advancing beyond current constraints through the prioritization of large-scale, multicenter RCTs with longer follow-up and standardized outcome measures is essential to generate higher-level evidence and strengthen the methodological rigor of the field.

#### 3.7.2 Core disease focus and expansion into multimorbidity

COPD was positioned as the absolute and central focus of this research domain. This was numerically affirmed: “chronic obstructive pulmonary disease” had the highest frequency (103), solidifying its role as the structural hub. The keyword “pulmonary rehabilitation” represented a key sub-field. Research in this area progressed from establishing efficacy to comparing innovative delivery models. Examples of such comparisons included home-based telerehabilitation versus traditional center-based programs (67, 68). Furthermore, “middle aged” and “disease progression” pointed to a concentrated research interest in the disease trajectory of the core patient demographic. Specifically, “disease progression” had the highest centrality (0.16) despite a moderate frequency (21), marking it as the critical structural bridge connecting pathology, intervention, and outcomes. Given the complex clinical scenarios presented by such patients, several major trials have been conducted on digital programs specifically tailored for patients with both COPD and chronic heart failure (CHF), or for the management of multiple chronic conditions (69–71). This demonstrated that the field was moving beyond a single disease focus to address the integrated management needs of the COPD population with multimorbidity.

#### 3.7.3 Integrating digital technology into COPD care delivery

The analysis identified a cohesive sub-network linking “remote monitoring data”, “ambulatory care”, and “medication adherence”, defining a leading edge in clinical research by modeling integrated care. This paradigm was operationalized through diverse and specific technological strategies: (1) Monitoring and Early Warning: deploying smartphones and wearables (72–74) for daily symptom and vital sign tracking to facilitate early detection of exacerbations. (2) Rehabilitation and Training: providing home-based telerehabilitation and exercise coaching via real-time video (75), gamification (76), or IoT-integrated systems (77). (3) Medication and Education: utilizing smart inhalers (78) to improve adherence, and conducting patient education through virtual visits (79) or AI chatbots (80). The keyword “time factors” underscored the importance of longitudinal engagement in these models. Furthermore, the high frequency and centrality of “quality of life” supported its role as an important patient-reported outcome linking technological efficacy with patient benefit (74). However, the effects of these technological paths were heterogeneous. While some interventions showed positive impacts on quality of life (81–84), exercise capacity (85, 86), or readmission rates (87, 88), some studies found no significant difference compared to usual care in reducing overall hospitalization (89, 90) or demonstrating cost-effectiveness (48, 91, 92). This inconsistency indicates that the clinical value of digital technology is highly dependent on the specific implementation, target population, and depth of integration with existing care services. The peripheral position of keywords like “health behavior” and “self-efficacy” further revealed a gap: despite advanced tools, theory-driven behavioral interventions remained underdeveloped, with few exceptions (93, 94). Thus, the next phase requires fusing technological innovation with behavioral science to achieve sustained and effective integrated care.

## 4 Discussion

### 4.1 Research hotspots and frontiers

This study identified and anticipated emerging trends in mHealth for COPD research by examining keyword bursts, citation bursts, and, in particular, the progress of clinical trials.

#### 4.1.1 Key mHealth and its core functional components

Currently, *remote monitoring and early warning systems*, *tele- and home-based rehabilitation*, s*elf-management support through mHealth*, and *smart medication adherence management* represent major functional components of this field.

*Remote monitoring and early warning systems* represent an important component of mHealth interventions. Their core function is the early identification of exacerbations through continuous, passive collection of physiological data. Traditionally, this relied on patients manually performing daily measurements, such as with fingertip pulse oximeters or home spirometers, and uploading the data (72, 95). More recently, the field has moved toward more seamless and integrated monitoring. Research confirms that incorporating smartwatches and patch-based sensors enables continuous tracking of oxygen saturation, respiratory rate, heart rate, and activity levels (96). Algorithms analyzing these data can automatically identify deterioration trends with significantly better sensitivity and specificity for predicting exacerbations than traditional symptom diaries (97, 98).

*Tele- and home-based rehabilitation* represents one of the most rapidly evolving mHealth modules, addressing the critical barrier of low accessibility to traditional center-based pulmonary rehabilitation. Early studies primarily established that home-based rehabilitation guided by phone or pre-recorded video was non-inferior to center-based programs in improving exercise capacity (e.g., 6-min walk distance) and quality of life (99, 100). The current frontier has advanced to real-time interactive telerehabilitation. Through high-definition video conferencing, therapists can provide live exercise instruction, adjust training intensity, and offer immediate feedback, with both efficacy and patient engagement supported by high-quality evidence (36, 37). Furthermore, the introduction of gamification and virtual reality technologies may enhance long-term adherence and exercise motivation by increasing the enjoyment and immersion of rehabilitation sessions, particularly for younger patients or those less engaged with traditional models (101, 102).

*Self-management support through mHealth* moves beyond simple information delivery to empower patients through structured education, cognitive behavioral therapy, and community support. Smartphone applications built on theoretical frameworks (e.g., Social Cognitive Theory, Theory of Planned Behavior) can deliver personalized symptom action plans, breathing technique training, and modules for managing anxiety (103). A randomized controlled trial demonstrated that telephone-delivered cognitive behavioral therapy significantly reduced depressive symptoms in patients with COPD (104). Recent evidence further supported the efficacy of digital cognitive behavior therapy (CBT) interventions in alleviating psychological distress and improving quality of life in this patient population (105). Meanwhile, AI-powered chatbots and online peer support communities offer scalable, low-cost avenues for continuous support, demonstrating clear benefits in improving patients’ disease knowledge and management confidence (98, 106).

*Smart medication adherence management* directly addresses a key challenge in COPD management. The central component, the smart inhaler, uses built-in sensors to record the time, dose, and technique of each use, syncing data to both patient and clinician dashboards (107). To address low medication adherence, digital interventions like smart inhalers offer an effective solution. When combined with reminders and feedback, they significantly improve medication-taking behavior. A recent meta-analysis found that such digital interventions increased the average medication adherence rate in COPD patients by 18% (108), and a systematic review noted that most studies showed a positive effect on medication adherence (109). Additionally, auxiliary tools such as augmented reality-based inhaler technique training (110) and pharmacist-led remote medication reviews (111) may help improve correct inhaler technique among patients, particularly older and newly diagnosed patients, thereby supporting effective drug delivery.

These core functional components do not exist in isolation but are increasingly converging into integrated care platforms. For example, the latest platforms can combine monitoring data from wearables, rehabilitation progress, medication records, and patient-reported outcomes into a single interface for shared decision-making (112).

#### 4.1.2 Target demographics and disease progression focus

Clinical trials in COPD typically recruit middle-aged to elderly patients with moderate-to-severe airflow limitation and an exacerbation history. This strategically targets the highest-burden population, ensuring research relevance. Real-world evidence robustly supports this focus, demonstrating that a prior exacerbation is a strong predictor of future exacerbations, hospitalizations, and mortality, thereby identifying the highest-risk patients. For instance, recent cohort studies confirm that patients classified as GOLD Group E, which reflects a high exacerbation-risk profile, have a significantly elevated risk of severe outcomes compared with those with milder disease (113, 114). However, this focus has also led to a notable gap: research on preventive digital interventions for early-stage COPD (GOLD 1) might be lacking (115). The potential of digital tools for risk factor management and behavior change in the early stages of the disease remains underexplored, probably limiting the perspective on their application across the entire disease continuum.

Despite aiming to delay “disease progression,” clinical effects are inconsistent. Some studies show potential in reducing exacerbations (90, 97, 98). However, several large, high-quality trials (e.g., PROMETE II, CHROMED) failed to demonstrate superiority of digital interventions over usual care in reducing hospitalization rates (53, 89, 90). Similarly, in a systematic review, Mishra et al. found mixed effects across 12 randomized controlled trials, with only 4 studies showing reduced readmission-related outcomes, suggesting that variation was related to intervention design, patient characteristics, and implementation context (116). More recent evidence further indicates that usability, acceptance, and user-centered design are important factors influencing the effective adoption of digital tools among older patients with COPD. Chien reported navigation and connectivity difficulties in 62% (11/18) of patients despite generally high satisfaction (117). Jiang et al. showed that patient decision aid–supported shared decision-making improved adherence and rehabilitation-related outcomes (118). Wegener et al. further found that older adults preferred optional, personalized digital functions that complemented rather than replaced clinician contact (119). Therefore, future mHealth studies should evaluate usability, acceptance, and design fit alongside clinical endpoints to better explain why apparently similar interventions yield different outcomes.

In response to the reality that COPD patients often have comorbidities such as heart failure and anxiety/depression, research has begun designing integrated care programs (62, 69–71). This explores the role of digital platforms in coordinating polypharmacy and managing complex conditions. However, comorbidities are currently often treated as factors for subgroup analysis rather than the core of intervention design. Developing digital management solutions centered on the patient’s overall health is crucial for the future.

Taken together, these findings suggest that the inconsistent outcomes observed across studies are likely related not only to differences in patient characteristics and implementation context, but also to limitations in the current evidence base (120). In particular, variation in intervention design, outcome assessment, and study scale makes it difficult to compare results across studies and draw firm conclusions. Therefore, future research should prioritize multicenter studies with larger sample sizes, longer follow-up, and more standardized outcome measures to strengthen the evidence base.

#### 4.1.3 Research frontiers and emerging hotspots

Artificial intelligence and machine learning represent the most active frontier. The research hotspot focuses on developing predictive models to identify the risk of COPD exacerbations in advance. For instance, studies construct “digital phenotypes” by analyzing vital signs from remote monitoring or passive data such as cough and activity patterns captured by smartphone sensors to achieve early warning (121, 122). Furthermore, adaptive algorithms are being explored for telerehabilitation, dynamically adjusting exercise prescriptions based on a patient’s daily condition, signifying an evolution from static programs to dynamic, personalized support (123).

IoT and wearable device technologies are driving the shift from fragmented interventions to integrated, continuous care models. The research hotspot is reflected in the use of devices like smartwatches and oximeters to continuously collect multidimensional physiological and activity data, providing an objective basis for assessment (124). In addition, intelligent medication management, such as sensor-equipped inhalers that objectively record medication use, forms an adherence feedback loop (125). A further emerging area aims to connect home ventilators and monitors via IoT platforms to construct a “virtual ward” for the out-of-hospital management of patients with severe COPD (126).

However, wider use of these technologies also requires attention to the risks associated with large-scale data collection. Although continuous monitoring and AI-assisted analysis may improve early risk identification and support more individualized intervention, they may also raise concerns regarding privacy and data security, variable data quality, false alerts, and insufficient clinical credibility when digital outputs are not adequately validated or interpreted alongside clinical assessment (127). Future implementation should therefore emphasize privacy safeguards, standardized data acquisition and quality control, age-appropriate design and support, and stronger clinical oversight.

#### 4.1.4 Geographical disparities and collaborative networks

The United States ranked first in publication output, indicating that it was the most productive national contributor and a major driver of research activity in mHealth research for COPD. England’s lead in total citations, together with the lasting influence of early UK mHealth trials and implementation-oriented studies (31, 33), suggests a particularly important foundational role in shaping subsequent COPD telehealth research. By contrast, Canada had the highest average citation count despite a lower publication volume, indicating relatively strong citation impact at the per-publication level. The institutional co-authorship network also showed a prominent collaboration between the University of Toronto and the University Health Network, indicating an active academic-clinical research connection in Canada.

The international collaboration network also showed a relatively centralized structure. Close collaborations frequently occur among top-tier universities, hospitals, and corporations within the core circle, such as Norway’s MILA project which integrated municipal health services, general practitioners, hospital specialists, and technology companies (128). While such alliances of strong entities can rapidly advance technological development and standard-setting, this model may inadvertently create knowledge barriers. It risks shaping research agendas, technical standards, and efficacy judgments according to the logic of high-resource settings. This overlooks the fact that in Low- and Middle-Income Countries (LMICs) with weak infrastructure and uneven digital literacy, feasibility, acceptability, and sustainability are the primary challenges (129, 130).

Notably, some forward-looking research is beginning to directly address this equity issue. The innovative path lies not in simply “transplanting” complex systems, but in developing “appropriate technologies” that closely match local resources. For instance, recent research utilizing smartphone microphones for AI-driven auscultation to screen for exacerbations was explicitly designed to provide a scalable (98), low-cost tool for regions lacking pulmonary specialists. Similarly, large multicenter studies led by high-burden countries (e.g., China) (131), aiming to build IoT-based intelligent management platforms, represent innovation driven from the demand side, which is more likely to produce locally adaptive solutions.

Therefore, promoting global research equity is not only an ethical imperative but also a strategic necessity to enhance the overall scientific value and practical impact of the field. Future efforts should focus on three priorities. First, demand-driven research led by LMICs should be supported, particularly studies validating low-tech interventions such as SMS reminders, simplified apps, and AI-assisted primary diagnosis (132). Second, global equitable partnerships should be strengthened by integrating technology and funding from core research groups with clinical insights and implementation experience from high-burden regions (133). Third, equity assessments should be incorporated at the research design stage, with digital accessibility and cultural adaptability used as key evaluation metrics (134). The ultimate goal is to ensure that the evidence generated can effectively serve and represent the world’s diverse patients and healthcare systems through a “decentralization” of the research ecosystem.

### 4.2 Strengths and limitations

This study adopted a parallel dual-database design and integrated multiple bibliometric tools, including CiteSpace, VOSviewer, and HistCite, to provide a comprehensive overview of publication trends, collaboration patterns, citation relationships, keyword evolution, clinical trial progress, and influential publications in mHealth research for COPD. The screening and reporting process followed the BIBLIO checklist to enhance methodological transparency.

However, several limitations should be acknowledged. To ensure consistency in bibliographic data structure and compatibility with the visualization tools, this study included only English-language articles, reviews, and clinical trials. This restriction may have led to the omission of some relevant non-English publications, especially studies conducted in non-English-speaking regions. Future research may further examine non-English literature to provide a more comprehensive understanding of this field.

In addition, although bibliometric analysis is well suited to mapping publication trends, research hotspots, citation relationships, and knowledge structures, citation-based or network-based indicators mainly reflect research activity and academic attention. These indicators should not be interpreted as direct evidence for the effectiveness or superiority of specific interventions (135). Therefore, the findings of this study should be understood as a structural overview of research development and thematic evolution in mHealth for COPD, rather than as a direct comparison of intervention efficacy.

In the clinical trial analysis, this study focused on mapping clinical research directions rather than pooling intervention effects, given the diversity of intervention types and outcome measures. Future studies may conduct quantitative synthesis for more homogeneous subsets of interventions, such as telemonitoring or telerehabilitation, when sufficient comparable evidence is available. Furthermore, because mHealth technologies and related clinical applications are rapidly evolving, the most recent technological applications and clinical findings may not have been fully captured. Maintaining active communication with frontline researchers, technology developers, and clinicians will therefore be important for tracking the latest developments and understanding the challenges and opportunities of real-world implementation.

## 5 Conclusion

This study methodically summarizes the current status, research hotspots, and emerging trends in the application of mHealth for COPD. The findings highlight the sustained growth in academic interest and the establishment of a core research paradigm focused on managing disease progression in high-risk patients. Future basic and clinical research should further validate whether digital tools can reduce key long-term outcomes, such as exacerbations and hospitalizations. Their application in comorbidity management also warrants further investigation. In terms of research methods, artificial intelligence predictive models, Internet of Things monitoring devices, and intervention designs integrated with behavioral science will become important tools for enhancing personalized management. This study also reveals significant geographical disparities, with most studies led by high-income countries. Therefore, the applicability of these findings to low- and middle-income countries, which bear the greatest disease burden, requires further validation. Addressing these disparities will require context-adapted digital interventions, stronger cross-regional collaboration, and implementation pathways that account for digital access, health literacy, and local health-system capacity. Promoting the equitable distribution of research resources and developing appropriate technologies suited to diverse healthcare environments are key to the field’s future broad impact.

## References

[1] Yappalparvi A Balaraman AK Padmapriya G Gaidhane S Kaur I Lal M . Safety and efficacy of ensifentrine in COPD: a systemic review and meta-analysis. Respir Med. (2025) 236:107863. doi: 10.1016/j.rmed.2024.107863, 39557208

[2] NCD Countdown 2030 collaborators. NCD countdown 2030: worldwide trends in non-communicable disease mortality and progress towards sustainable development goal target 3.4. Lancet. (2018) 392:1072–88. doi: 10.1016/S0140-6736(18)31992-5, 30264707

[3] Venkatesan P. GOLD COPD report: 2024 update. Lancet Respir Med. (2024) 12:15–6. doi: 10.1016/S2213-2600(23)00461-7, 38061380

[4] Hidalgo Sierra V Hernández Mezquita MÁ Palomo Cobos L García Sánchez M Castellanos RD Jodra Sánchez S . Usefulness of the Piko-6 portable device for early COPD detection in primary care. Arch Bronconeumol (Engl Ed). (2018) 54:460–6. doi: 10.1016/j.arbres.2018.04.015, 29880313

[5] Cozad MJ Crum M Tyson H Fleming PR Stratton J Kennedy AB . Mobile health apps for patient-Centered care: review of United States rheumatoid arthritis apps for engagement and activation. JMIR Mhealth Uhealth. (2022) 10:e39881. doi: 10.2196/39881, 36469397 PMC9764152

[6] McLean S Protti D Sheikh A. Telehealthcare for long term conditions. BMJ. (2011) 342:d120. doi: 10.1136/bmj.d120, 21292710

[7] Janjua S Carter D Threapleton CJ Prigmore S Disler RT. Telehealth interventions: remote monitoring and consultations for people with chronic obstructive pulmonary disease (COPD). Cochrane Database Syst Rev. (2021) 2021:CD013196. doi: 10.1002/14651858.CD013196.pub2, 34693988 PMC8543678

[8] Cox NS Dal Corso S Hansen H McDonald CF Hill CJ Zanaboni P . Telerehabilitation for chronic respiratory disease. Cochrane Database Syst Rev. (2021) 2021:CD013040. doi: 10.1002/14651858.CD013040.pub2, 33511633 PMC8095032

[9] Alwashmi MF Fitzpatrick B Farrell J Gamble JM Davis E Nguyen HV . Perceptions of patients regarding mobile health interventions for the management of chronic obstructive pulmonary disease: mixed methods study. JMIR Mhealth Uhealth. (2020) 8:e17409. doi: 10.2196/17409, 32706697 PMC7413289

[10] Jacob C Sanchez-Vazquez A Ivory C. Social, organizational, and technological factors impacting clinicians' adoption of mobile health tools: systematic literature review. JMIR Mhealth Uhealth. (2020) 8:e15935. doi: 10.2196/15935, 32130167 PMC7059085

[11] Tian J Dong YX Wang L Wu YM Zhao ZY Che GW. Mapping the evolution of 3D printing in cardio-thoracic diseases: a global bibliometric analysis. Int J Surg. (2025) 111:1629–35. doi: 10.1097/JS9.0000000000002095, 39352114 PMC11745706

[12] Park HY Kong S Lee M Ryu H Hamakawa Y Luppi F . Digital health technologies for improving the management of people with chronic obstructive pulmonary disease. Front Digit Health. (2025) 7:1640585. doi: 10.3389/fdgth.2025.1640585, 40843141 PMC12365816

[13] Xu H Jiang X Zeng Q Li R. Application of e-health tools in the assessment of inhalation therapy adherence in patients with chronic obstructive pulmonary disease: scoping review coupled with bibliometric analysis. Respir Med. (2025) 236:107898. doi: 10.1016/j.rmed.2024.107898, 39638011

[14] Lacasse Y Casaburi R Sliwinski P Chaouat A Fletcher E Haidl P . Home oxygen for moderate hypoxaemia in chronic obstructive pulmonary disease: a systematic review and meta-analysis. Lancet Respir Med. (2022) 10:1029–37. doi: 10.1016/S2213-2600(22)00179-5, 35817074

[15] Adeloye D Song P Zhu Y Campbell H Sheikh A Rudan I . Global, regional, and national prevalence of, and risk factors for, chronic obstructive pulmonary disease (COPD) in 2019: a systematic review and modelling analysis. Lancet Respir Med. (2022) 10:447–58. doi: 10.1016/S2213-2600(21)00511-7, 35279265 PMC9050565

[16] Romiti GF Corica B Pipitone E Vitolo M Raparelli V Basili S . Prevalence, management and impact of chronic obstructive pulmonary disease in atrial fibrillation: a systematic review and meta-analysis of 4,200,000 patients. Eur Heart J. (2021) 42:3541–54. doi: 10.1093/eurheartj/ehab453, 34333599

[17] Sîrbu V David OA. Efficacy of app-based mobile health interventions for stress management: a systematic review and meta-analysis of self-reported, physiological, and neuroendocrine stress-related outcomes. Clin Psychol Rev. (2024) 114:102515. doi: 10.1016/j.cpr.2024.102515, 39522422

[18] Suchodolska G Senkus E. Mobile applications for early breast cancer chemotherapy-related symptoms reporting and management: a scoping review. Cancer Treat Rev. (2022) 105:102364. doi: 10.1016/j.ctrv.2022.102364, 35231871

[19] Moon K Sobolev M Kane JM. Digital and mobile health technology in collaborative behavioral health care: scoping review. JMIR Ment Health. (2022) 9:e30810. doi: 10.2196/30810, 35171105 PMC8892315

[20] Thompson D Rattu S Tower J Egerton T Francis J Merolli M. Mobile app use to support therapeutic exercise for musculoskeletal pain conditions may help improve pain intensity and self-reported physical function: a systematic review. J Physiother. (2023) 69:23–34. doi: 10.1016/j.jphys.2022.11.012, 36528508

[21] Su Y Su X Chen Z Wang L Chen J. Bibliometric and visual analysis of gut microbiota research in functional bowel disorders from 2016 to 2025. Front Med (Lausanne). (2026) 13:1735121. doi: 10.3389/fmed.2026.1735121, 41684920 PMC12891093

[22] van Eck NJ Waltman L. Software survey: VOSviewer, a computer program for bibliometric mapping. Scientometrics. (2010) 84:523–38. doi: 10.1007/s11192-009-0146-3, 20585380 PMC2883932

[23] Hou J Du K Li J Li Z Cao S Zhang S . Research trends in the use of nanobodies for cancer therapy. J Control Release. (2025) 381:113454. doi: 10.1016/j.jconrel.2025.01.045, 39922288

[24] Tan L Wang X Yuan K Yin T Du R Shen L . Structural and temporal dynamics analysis on drug-eluting stents: history, research hotspots and emerging trends. Bioact Mater. (2023) 23:170–86. doi: 10.1016/j.bioactmat.2022.09.009, 36406256 PMC9663333

[25] World Health Organization. Chronic Obstructive Pulmonary Disease (COPD). (2024). Available online at: https://www.who.int/news-room/fact-sheets/detail/chronic-obstructive-pulmonary-disease-%28copd%29 (Accessed February 4, 2026).

[26] Wei W Ge J Xu S Li M Zhao Z Li X . Knowledge maps of disaster medicine in China based on co-word analysis. Disaster Med Public Health Prep. (2019) 13:405–9. doi: 10.1017/dmp.2018.63, 30033890

[27] Wootton R. Twenty years of telemedicine in chronic disease management--an evidence synthesis. J Telemed Telecare. (2012) 18:211–20. doi: 10.1258/jtt.2012.120219, 22674020 PMC3366107

[28] Sanders C Rogers A Bowen R Bower P Hirani S Cartwright M . Exploring barriers to participation and adoption of telehealth and telecare within the whole system demonstrator trial: a qualitative study. BMC Health Serv Res. (2012) 12:220. doi: 10.1186/1472-6963-12-220, 22834978 PMC3413558

[29] Kuijpers W Groen WG Aaronson NK van Harten WH. A systematic review of web-based interventions for patient empowerment and physical activity in chronic diseases: relevance for cancer survivors. J Med Internet Res. (2013) 15:e37. doi: 10.2196/jmir.2281, 23425685 PMC3636300

[30] Johnston B Wheeler L Deuser J Sousa KH. Outcomes of the Kaiser Permanente Tele-home Health Research project. Arch Fam Med. (2000) 9:40–5. doi: 10.1001/archfami.9.1.40, 10664641

[31] Pinnock H Hanley J McCloughan L Todd A Krishan A Lewis S . Effectiveness of telemonitoring integrated into existing clinical services on hospital admission for exacerbation of chronic obstructive pulmonary disease: researcher blind, multicentre, randomised controlled trial. BMJ. (2013) 347:f6070. doi: 10.1136/bmj.f6070, 24136634 PMC3805483

[32] Bashshur RL Shannon GW Smith BR Alverson DC Antoniotti N Barsan WG . The empirical foundations of telemedicine interventions for chronic disease management. Telemed J E Health. (2014) 20:769–800. doi: 10.1089/tmj.2014.9981, 24968105 PMC4148063

[33] Steventon A Bardsley M Billings J Dixon J Doll H Hirani S . Effect of telehealth on use of secondary care and mortality: findings from the whole system demonstrator cluster randomised trial. BMJ. (2012) 344:e3874. doi: 10.1136/bmj.e3874, 22723612 PMC3381047

[34] Polisena J Tran K Cimon K Hutton B McGill S Palmer K . Home telehealth for chronic obstructive pulmonary disease: a systematic review and meta-analysis. J Telemed Telecare. (2010) 16:120–7. doi: 10.1258/jtt.2009.090812, 20197355

[35] Vitacca M Bianchi L Guerra A Fracchia C Spanevello A Balbi B . Tele-assistance in chronic respiratory failure patients: a randomised clinical trial. Eur Respir J. (2009) 33:411–8. doi: 10.1183/09031936.00005608, 18799512

[36] Holland AE Cox NS Houchen-Wolloff L Rochester CL Garvey C ZuWallack R . Defining modern pulmonary rehabilitation. An official American Thoracic Society workshop report. Ann Am Thorac Soc. (2021) 18:e12–29. doi: 10.1513/AnnalsATS.202102-146ST, 33929307 PMC8086532

[37] GBD Chronic Respiratory Disease Collaborators. Prevalence and attributable health burden of chronic respiratory diseases, 1990–2017: a systematic analysis for the Global Burden of Disease Study 2017. Lancet Respir Med. (2020) 8:585–596. doi: 10.1016/S2213-2600(20)30105-3 32526187 PMC7284317

[38] Lundell S Holmner Å Rehn B Nyberg A Wadell K. Telehealthcare in COPD: a systematic review and meta-analysis on physical outcomes and dyspnea. Respir Med. (2015) 109:11–26. doi: 10.1016/j.rmed.2014.10.008, 25464906

[39] Bolton CE Waters CS Peirce S Elwyn G. Insufficient evidence of benefit: a systematic review of home telemonitoring for COPD. J Eval Clin Pract. (2011) 17:1216–22. doi: 10.1111/j.1365-2753.2010.01536.x, 20846317

[40] Koff PB Jones RH Cashman JM Voelkel NF Vandivier RW. Proactive integrated care improves quality of life in patients with COPD. Eur Respir J. (2009) 33:1031–8. doi: 10.1183/09031936.00063108, 19129289

[41] Cruz J Brooks D Marques A. Home telemonitoring in COPD: a systematic review of methodologies and patients' adherence. Int J Med Inform. (2014) 83:249–63. doi: 10.1016/j.ijmedinf.2014.01.008, 24529402

[42] Ure J Pinnock H Hanley J Kidd G McCall Smith E Tarling A . Piloting tele-monitoring in COPD: a mixed methods exploration of issues in design and implementation. Prim Care Respir J. (2012) 21:57–64. doi: 10.4104/pcrj.2011.00065, 21785816 PMC6548305

[43] Tabak M Brusse-Keizer M van der Valk P Hermens H Vollenbroek-Hutten M. A telehealth program for self-management of COPD exacerbations and promotion of an active lifestyle: a pilot randomized controlled trial. Int J Chron Obstruct Pulmon Dis. (2014) 9:935–44. doi: 10.2147/COPD.S60179, 25246781 PMC4166347

[44] Segrelles Calvo G Gómez-Suárez C Soriano JB Zamora E Gónzalez-Gamarra A González-Béjar M . A home telehealth program for patients with severe COPD: the PROMETE study. Respir Med. (2014) 108:453–62. doi: 10.1016/j.rmed.2013.12.003, 24433744

[45] Stickland M Jourdain T Wong EY Rodgers WM Jendzjowsky NG Macdonald GF. Using telehealth technology to deliver pulmonary rehabilitation in chronic obstructive pulmonary disease patients. Can Respir J. (2011) 18:216–20. doi: 10.1155/2011/640865, 22059179 PMC3205102

[46] Cruz J Brooks D Marques A. Home telemonitoring effectiveness in COPD: a systematic review. Int J Clin Pract. (2014) 68:369–78. doi: 10.1111/ijcp.12345, 24472009

[47] Dinesen B Haesum LK Soerensen N Nielsen C Grann O Hejlesen O . Using preventive home monitoring to reduce hospital admission rates and reduce costs: a case study of telehealth among chronic obstructive pulmonary disease patients. J Telemed Telecare. (2012) 18:221–5. doi: 10.1258/jtt.2012.110704, 22653618

[48] McDowell JE McClean S FitzGibbon F Tate S. A randomised clinical trial of the effectiveness of home-based health care with telemonitoring in patients with COPD. J Telemed Telecare. (2015) 21:80–7. doi: 10.1177/1357633X14566575, 25586812

[49] Chau JP Lee DT Yu DS Chow AY Yu WC Chair SY . A feasibility study to investigate the acceptability and potential effectiveness of a telecare service for older people with chronic obstructive pulmonary disease. Int J Med Inform. (2012) 81:674–82. doi: 10.1016/j.ijmedinf.2012.06.003 22789911

[50] Demeyer H Louvaris Z Frei A Rabinovich RA de Jong C Gimeno-Santos E . Physical activity is increased by a 12-week semiautomated telecoaching programme in patients with COPD: a multicentre randomised controlled trial. Thorax. (2017) 72:415–23. doi: 10.1136/thoraxjnl-2016-209026, 28137918 PMC5520265

[51] Antoniades NC Rochford PD Pretto JJ Pierce RJ Gogler J Steinkrug J . Pilot study of remote telemonitoring in COPD. Telemed J E-Health. (2012) 18:634–40. doi: 10.1089/tmj.2011.0231, 22957501

[52] Williams V Price J Hardinge M Tarassenko L Farmer A. Using a mobile health application to support self-management in COPD: a qualitative study. Br J Gen Pract. (2014) 64:e392–400. doi: 10.3399/bjgp14X680473, 24982491 PMC4073724

[53] Walker PP Pompilio PP Zanaboni P Bergmo TS Prikk K Malinovschi A . Telemonitoring in chronic obstructive pulmonary disease (CHROMED). A randomized clinical trial. Am J Respir Crit Care Med. (2018) 198:620–8. doi: 10.1164/rccm.201712-2404OC, 29557669

[54] Ringbæk T Green A Laursen LC Frausing E Brøndum E Ulrik CS. Effect of tele health care on exacerbations and hospital admissions in patients with chronic obstructive pulmonary disease: a randomized clinical trial. Int J Chron Obstruct Pulmon Dis. (2015) 10:1801–8. doi: 10.2147/COPD.S85596, 26366072 PMC4562759

[55] Fairbrother P Pinnock H Hanley J McCloughan L Sheikh A Pagliari C . Continuity, but at what cost? The impact of telemonitoring COPD on continuities of care: a qualitative study. Prim Care Respir J. (2012) 21:322–8. doi: 10.4104/pcrj.2012.00068, 22875143 PMC6547965

[56] Barbosa MT Sousa CS Morais-Almeida M Simões MJ Mendes P. Telemedicine in COPD: an overview by topics. COPD: J Chron Obstruct Pulmon Dis. (2020) 17:601–17. doi: 10.1080/15412555.2020.1815182, 32892650

[57] Sorknaes AD Madsen H Hallas J Jest P Hansen‐Nord M. Nurse tele-consultations with discharged COPD patients reduce early readmissions--an interventional study. Clin Respir J. (2011) 5:26–34. doi: 10.1111/j.1752-699X.2010.00187.x 21159138

[58] Chatwin M Hawkins G Panicchia L Woods A Hanak A Lucas R . Randomised crossover trial of telemonitoring in chronic respiratory patients (TeleCRAFT trial). Thorax. (2016) 71:305–11. doi: 10.1136/thoraxjnl-2015-207045, 26962013 PMC4819626

[59] Sorknaes AD Bech M Madsen H Titlestad IL Hounsgaard L Hansen-Nord M . The effect of real-time teleconsultations between hospital-based nurses and patients with severe COPD discharged after an exacerbation. J Telemed Telecare. (2013) 19:466–74. doi: 10.1177/1357633X13512067, 24227799

[60] Lewis KE Annandale JA Warm DL Hurlin C Lewis MJ Lewis L. Home telemonitoring and quality of life in stable, optimised chronic obstructive pulmonary disease. J Telemed Telecare. (2010) 16:253–9. doi: 10.1258/jtt.2009.090907, 20483881

[61] Fairbrother P Pinnock H Hanley J McCloughan L Sheikh A Pagliari C . Exploring telemonitoring and self-management by patients with chronic obstructive pulmonary disease: a qualitative study embedded in a randomized controlled trial. Patient Educ Couns. (2013) 93:403–10. doi: 10.1016/j.pec.2013.04.003 23647981

[62] Whitten P Mickus M. Home telecare for COPD/CHF patients: outcomes and perceptions. J Telemed Telecare. (2007) 13:69–73. doi: 10.1258/135763307780096249, 17359569

[63] Liu WT Wang CH Lin HC Lin SM Lee KY Lo YL . Efficacy of a cell phone-based exercise programme for COPD. Eur Respir J. (2008) 32:651–9. doi: 10.1183/09031936.00104407, 18508824

[64] Goldstein RS O’Hoski S. Telemedicine in COPD: time to pause. Chest. (2014) 145:945–9. doi: 10.1378/chest.13-1656, 24798834

[65] Pedone C Chiurco D Scarlata S Incalzi RA. Efficacy of multiparametric telemonitoring on respiratory outcomes in elderly people with COPD: a randomized controlled trial. BMC Health Serv Res. (2013) 13:82. doi: 10.1186/1472-6963-13-82, 23497109 PMC3680224

[66] Karlsson Å Sönnerfors P Lundell S Toots A Wadell K. Evaluation of a novel eHealth tool for pulmonary rehabilitation in people with chronic obstructive pulmonary disease: randomized controlled pilot and feasibility trial. JMIR Form Res. (2025) 9:e68195. doi: 10.2196/68195, 40550122 PMC12235207

[67] Tsai LL McNamara RJ Moddel C Tsai LLY Alison JA McKenzie DK . Home-based telerehabilitation via real-time videoconferencing improves endurance exercise capacity in patients with COPD: the randomized controlled TeleR study. Respirology. (2017) 22:699–707. doi: 10.1111/resp.12966, 27992099

[68] Hou Y Wang Y Huang X Sun M Nan J Gao J . A feasibility study of an autonomy-supportive intervention in remote pulmonary rehabilitation for older adults with chronic obstructive pulmonary disease. Age Ageing. (2025) 54:afaf282. doi: 10.1093/ageing/afaf282, 41066673

[69] Harvey BP Barenfeld E Fors A Ekman I Swedberg K Gyllensten H. Economic evaluation of a person-centred care intervention with a digital platform and structured telephone support for people with chronic heart failure and/or chronic obstructive pulmonary disease: results from a randomised controlled trial in Sweden. BMJ Open. (2025) 15:e093083. doi: 10.1136/bmjopen-2024-093083, 41067758 PMC12516990

[70] Whelan ME Velardo C Rutter H Tarassenko L Farmer AJ. Mood monitoring over one year for people with chronic obstructive pulmonary disease using a mobile health system: retrospective analysis of a randomized controlled trial. JMIR Mhealth Uhealth. (2019) 7:e14946. doi: 10.2196/14946, 31755872 PMC6898889

[71] Jiang W Song Y. Internet of things-based home noninvasive ventilation in COPD patients with hypercapnic chronic respiratory failure: study protocol for a randomized controlled trial. Trials. (2022) 23:393. doi: 10.1186/s13063-022-06372-z, 35551646 PMC9097410

[72] Püschner F Schiller J Urbanski-Rini D Scholl K Bock A Jandl M . TELEMEdizinisches moNiTORing für COPD-Patienten (Telementor COPD): Studienprotokoll einer multizentrischen, randomisierten, kontrollierten Studie [TELEMEdical moNiTORing for COPD patients (Telementor COPD): study protocol of a multicentre, randomised, controlled study]. Pneumologie. (2025) 79:358–65. doi: 10.1055/a-2383-4470, 39208875 PMC12068928

[73] Hill K Ng LWC Cecins N Formico VR Cavalheri V Jenkins SC. Effect of using a wheeled walker on physical activity and sedentary time in people with chronic obstructive pulmonary disease: a randomised cross-over trial. Lung. (2020) 198:213–9. doi: 10.1007/s00408-019-00297-2 31828516

[74] Wang L Guo Y Wang M Zhao Y. A mobile health application to support self-management in patients with chronic obstructive pulmonary disease: a randomised controlled trial. Clin Rehabil. (2021) 35:90–101. doi: 10.1177/0269215520946931 32907384

[75] Nield M Hoo GW. Real-time telehealth for COPD self-management using skype™. COPD. (2012) 9:611–9. doi: 10.3109/15412555.2012.708067, 22946768

[76] Jiang Y Sun M Nuerdawulieti B Huang X Hou Y Nan J . Effectiveness of remote gamification pulmonary rehabilitation intervention based on the health action process approach theory in older adults with chronic obstructive pulmonary disease: a pilot randomized controlled trial. Front Med (Lausanne). (2025) 12:1576256. doi: 10.3389/fmed.2025.1576256, 40612580 PMC12221897

[77] Hosain MN Kwak YS Lee J Choi H Park J Kim J. IoT-enabled biosensors for real-time monitoring and early detection of chronic diseases. Phys Act Nutr. (2024) 28:60–9. doi: 10.20463/pan.2024.0033, 39934631 PMC11811615

[78] Cuperus LJA van der Palen J Aldenkamp A van Huisstede A Bischoff EWMA van Boven JFM . Adherence to single inhaler triple therapy and digital inhalers in chronic obstructive pulmonary disease: a literature review and protocol for a randomized controlled trial (TRICOLON study). BMC Pulm Med. (2024) 24:317. doi: 10.1186/s12890-024-03044-3, 38965541 PMC11225120

[79] Press VG Arora VM Kelly CA Carey KA White SR Wan W. Effectiveness of virtual vs in-person inhaler education for hospitalized patients with obstructive lung disease: a randomized clinical trial. JAMA Netw Open. (2020) 3:e1918205. doi: 10.1001/jamanetworkopen.2019.18205, 31899529 PMC6991242

[80] Merç P Pirinççi CŞ Cihan E. Evaluation of AI chatbots for patient education and information on chronic obstructive pulmonary disease. Heart Lung. (2026 Jan-Feb) 75:21–5. doi: 10.1016/j.hrtlng.2025.09.002, 40939398

[81] Glynn L Moloney E Lane S McNally E Buckley C McCann M . A smartphone app self-management program for chronic obstructive pulmonary disease: randomized controlled trial of clinical outcomes. JMIR Mhealth Uhealth. (2025) 13:e56318. doi: 10.2196/56318, 40267465 PMC12059498

[82] Kronborg T Hangaard S Laursen SH Hæsum LKE Egmose J Bender C . Impact of telemonitoring with exacerbation prediction algorithm versus telemonitoring alone on hospitalizations and health-related quality of life in patients with COPD. Respir Care. (2025) 70:954–61. doi: 10.1089/respcare.12611, 40192545 PMC12411414

[83] Naranjo-Rojas A de Perula- Torres LÁ Cruz-Mosquera FE Molina-Recio G. Efficacy and acceptability of a mobile app for monitoring the clinical status of patients with chronic obstructive pulmonary disease receiving home oxygen therapy: randomized controlled trial. J Med Internet Res. (2025) 27:e65888. doi: 10.2196/65888, 39761550 PMC11747540

[84] Spielmanns M Gloeckl R Jarosch I Leitl D Schneeberger T Boeselt T . Using a smartphone application maintains physical activity following pulmonary rehabilitation in patients with COPD: a randomised controlled trial. Thorax. (2023) 78:442–50. doi: 10.1136/thoraxjnl-2021-218338, 35450945 PMC10176348

[85] Vorrink SN Kort HS Troosters T Lammers J-WJ. A mobile phone app to stimulate daily physical activity in patients with chronic obstructive pulmonary disease: development, feasibility, and pilot studies. JMIR Mhealth Uhealth. (2016) 4:e11. doi: 10.2196/mhealth.4741, 26813682 PMC4748139

[86] Arbillaga-Etxarri A Gimeno-Santos E Barberan-Garcia A Balcells E Benet M Borrell E . Long-term efficacy and effectiveness of a behavioural and community-based exercise intervention (urban training) to increase physical activity in patients with COPD: a randomised controlled trial. Eur Respir J. (2018) 52:1800063. doi: 10.1183/13993003.00063-2018, 30166322 PMC6203405

[87] Andersen FD Trolle C Pedersen AR Køpfli ML Børgesen S Jensen MS . Effect of telemonitoring on readmissions for acute exacerbation of chronic obstructive pulmonary disease: a randomized clinical trial. J Telemed Telecare. (2024) 30:1417–24. doi: 10.1177/1357633X221150279 36683440

[88] Köksal N Durgun H. Impact of telecounselling, home monitoring and exercise on hospital readmissions and quality of life in chronic obstructive pulmonary disease: a randomized controlled trial. Int J Nurs Pract. (2025) 31:e70021. doi: 10.1111/ijn.70021, 40387292 PMC12087424

[89] Soriano JB García-Río F Vázquez-Espinosa E Conforto JI Hernando-Sanz A López-Yepes L . A multicentre, randomized controlled trial of telehealth for the management of COPD. Respir Med. (2018) 144:74–81. doi: 10.1016/j.rmed.2018.10.008, 30366588

[90] Hyldgaard C Ringbæk T Andersen FD Andersen F Hansen E Jensen M . Effect of telemonitoring on moderate and severe exacerbations in patients with COPD: pooled analysis of two randomized controlled trials in Denmark. Int J Chron Obstruct Pulmon Dis. (2025) 20:2361–9. doi: 10.2147/COPD.S528852, 40666254 PMC12262086

[91] Bentley CL Mountain GA Thompson J Fitzsimmons DA Lowrie K Parker SG . A pilot randomised controlled trial of a telehealth intervention in patients with chronic obstructive pulmonary disease: challenges of clinician-led data collection. Trials. (2014) 15:313. doi: 10.1186/1745-6215-15-313, 25100550 PMC4131041

[92] Witt Udsen F Lilholt PH Hejlesen O Ehlers L. Cost-effectiveness of telehealthcare to patients with chronic obstructive pulmonary disease: results from the Danish 'TeleCare north' cluster-randomised trial. BMJ Open. (2017) 7:e014616. doi: 10.1136/bmjopen-2016-014616, 28515193 PMC5541337

[93] Fors A Blanck E Ali L Ekberg-Jansson A Fu M Lindström Kjellberg I . Effects of a person-centred telephone-support in patients with chronic obstructive pulmonary disease and/or chronic heart failure - a randomized controlled trial. PLoS One. (2018) 13:e0203031. doi: 10.1371/journal.pone.0203031, 30169539 PMC6118377

[94] Cho YM Lee S Islam SMS Kim SY. Theories applied to m-health interventions for behavior change in low- and middle-income countries: a systematic review. Telemed J E Health. (2018) 24:727–41. doi: 10.1089/tmj.2017.0249, 29437546 PMC6205046

[95] Hu C Liao X Fang Y Zhu S Lan X Cheng G. Clinical and cost-effectiveness of telehealth-supported home oxygen therapy on adherence, hospital readmission, and health-related quality of life in patients with chronic obstructive pulmonary disease: systematic review and meta-analysis of randomized controlled trials. J Med Internet Res. (2025) 27:e73010. doi: 10.2196/73010, 40631803 PMC12262104

[96] Iorio OC Coutu FA Malaeb D Ross BA. Feasibility, functionality, and user experience with wearable technologies for acute exacerbation monitoring in patients with severe COPD. Front Signal Process. (2024) 4:1362754. doi: 10.3389/frsip.2024.1362754

[97] Hatipoğlu U. Telemonitoring to reduce COPD exacerbations: a work in progress. Respir Care. (2025) 70:1067–8. doi: 10.1089/respcare.13121, 40340617 PMC12411403

[98] Gong Y Xu C Mo C Wu J Lin H Su H . AI-driven smartphone screening for acute COPD exacerbations: enhancing health equity in developing regions. NPJ Digit Med. (2025) 8:715. doi: 10.1038/s41746-025-02086-z, 41272082 PMC12638963

[99] Godtfredsen N Frølich A Bieler T Beyer N Kallemose T Wilcke T . 12-months follow-up of pulmonary tele-rehabilitation versus standard pulmonary rehabilitation: a multicentre randomised clinical trial in patients with severe COPD. Respir Med. (2020) 172:106129. doi: 10.1016/j.rmed.2020.106129, 32905893

[100] Cox NS McDonald C Burge AT Hill CJ Bondarenko J Holland AE. Comparison of clinically meaningful improvements after center-based and home-based telerehabilitation in people with COPD. Chest. (2025) 167:1003–11. doi: 10.1016/j.chest.2024.11.001 39522594

[101] Patsaki I Avgeri V Rigoulia T Zekis T Koumantakis GA Grammatopoulou E. Benefits from incorporating virtual reality in pulmonary rehabilitation of COPD patients: a systematic review and meta-analysis. Adv Respir Med. (2023) 91:324–36. doi: 10.3390/arm91040026, 37622840 PMC10451922

[102] Chen J Yang T He Q Pang M Cao Y Liu Z . The impact of gamified interventions on the management of chronic obstructive pulmonary disease: systematic literature review. JMIR Serious Games. (2025) 13:e69510. doi: 10.2196/69510, 40446290 PMC12166322

[103] Chen Y Tan R Long X Tu H. Applying behavioral change theories to optimize pulmonary rehabilitation in COPD patients: a review. Medicine (Baltimore). (2024) 103:e38366. doi: 10.1097/MD.0000000000038366, 39259106 PMC11142794

[104] Doyle C Bhar S Fearn M Ames D Osborne D You E . The impact of telephone-delivered cognitive behaviour therapy and befriending on mood disorders in people with chronic obstructive pulmonary disease: a randomized controlled trial. Br J Health Psychol. (2017) 22:542–56. doi: 10.1111/bjhp.12245 28544504

[105] Yuan L Yang Q Li Y Li L Deng S Li S. Effect of computerized cognitive behavioral therapy on symptom cluster management in patients with chronic obstructive pulmonary disease: a randomized controlled trial. J Tuberc Lung Dis. (2024) 5:476–83. doi: 10.19983/j.issn.2096-8493.2024117

[106] Malanga E Malanga V Latham S. Websites, online communities and digital channels as a medium to effectively educate, engage and empower patients. Chronic Obstr Pulm Dis. (2018) 5:334–7. doi: 10.15326/jcopdf.5.4.2018.0158, 30723789 PMC6361474

[107] Jansen EM van de Hei SJ Dierick BJH Kerstjens HAM Kocks JWH van Boven JFM. Global burden of medication non-adherence in chronic obstructive pulmonary disease (COPD) and asthma: a narrative review of the clinical and economic case for smart inhalers. J Thorac Dis. (2021) 13:3846–64. doi: 10.21037/jtd-20-2360, 34277075 PMC8264677

[108] Aung H Tan R Flynn C Divall P Wright A Murphy A . Digital remote maintenance inhaler adherence interventions in COPD: a systematic review and meta-analysis. Eur Respir Rev. (2024) 33:240136. doi: 10.1183/16000617.0136-2024, 39631930 PMC11615661

[109] Muruganantham S Ramesh SL. Soft mist, dry powder, and smart inhalers: comparative technologies and clinical impact on asthma and COPD management. Egypt J Bronchol. (2025) 19:109. doi: 10.1186/s43168-025-00461-8

[110] Chai X Wu L He Z. Effects of virtual reality-based pulmonary rehabilitation in patients with chronic obstructive pulmonary disease: a meta-analysis. Medicine (Baltimore). (2023) 102:e36702. doi: 10.1097/MD.0000000000036702, 38206693 PMC10754576

[111] Sarwar MR McDonald VM Abramson MJ Wilson S Holland AE Bonevski B . Credentialed pharmacist-led home medicines reviews targeting treatable traits and their impact on health outcomes in people with chronic obstructive pulmonary disease: a pre- and post-intervention study. Int J Clin Pharm. (2025) 47:157–65. doi: 10.1007/s11096-024-01819-6, 39466489 PMC11742330

[112] Huang X Song X Sun M Hou Y Nan J Gao J . Effectiveness of digital co-creation platform in remote pulmonary rehabilitation for older adults with chronic obstructive pulmonary disease: a randomized controlled trial. Front Public Health. (2025) 13:1708607. doi: 10.3389/fpubh.2025.1708607, 41293596 PMC12640875

[113] Lin L Song Q Cheng W Li T Zhang P Liu C . Impact of exacerbation history on future risk and treatment outcomes in chronic obstructive pulmonary disease patients: a prospective cohort study based on global initiative for chronic obstructive lung disease (GOLD) a and B classifications. J Glob Health. (2024) 14:04202. doi: 10.7189/jogh.14.04202, 39388682 PMC11466499

[114] Waeijen-Smit K Peerlings DEM Jörres RA Watz H Bals R Rabe KF . GOLD COPD exacerbation history categories and disease outcomes. JAMA Netw Open. (2024) 7:e2445488. doi: 10.1001/jamanetworkopen.2024.45488, 39693071 PMC11656261

[115] Althobiani MA Russell AM Jacob J Ranjan Y Ahmad R Folarin AA . The role of digital health in respiratory diseases management: a narrative review of recent literature. Front Med (Lausanne). (2025) 12:1361667. doi: 10.3389/fmed.2025.1361667, 40078397 PMC11896871

[116] Mishra V Stuckler D McNamara CL. Digital interventions to reduce hospitalization and hospital readmission for chronic obstructive pulmonary disease (COPD) patient: systematic review. BMC Digit Health. (2024) 2:46. doi: 10.1186/s44247-024-00103-x

[117] Chien SY. Mobile app for patients with chronic obstructive pulmonary diseases during home-based exercise care: usability study. JMIR Hum Factors. (2024) 11:e60049. doi: 10.2196/60049, 39546767 PMC11607552

[118] Jiang Y Nuerdawulieti B Chen Z Guo J Sun P Chen M . Effectiveness of patient decision aid supported shared decision-making intervention in in-person and virtual hybrid pulmonary rehabilitation in older adults with chronic obstructive pulmonary disease: a pilot randomized controlled trial. J Telemed Telecare. (2024) 30:1532–42. doi: 10.1177/1357633X231156631, 36919365

[119] Wegener EK Bergschöld JM Kramer T Schmidt CW Borgnakke K. Co-designing a conversational agent with older adults with chronic obstructive pulmonary disease who age in place: qualitative study. JMIR Hum Factors. (2024) 11:e63222. doi: 10.2196/63222, 39378067 PMC11496918

[120] Zhuang M Hassan W II Ahmad WMA Abdul Kadir A Liu X Li F . Effectiveness of digital health interventions for chronic obstructive pulmonary disease: systematic review and meta-analysis. J Med Internet Res. (2025) 27:e76323. doi: 10.2196/76323, 40418567 PMC12149779

[121] Snyder LD DePietro M Reich M Neely ML Lugogo N Pleasants R . Predictive machine learning algorithm for COPD exacerbations using a digital inhaler with integrated sensors. BMJ Open Respir Res. (2025) 12:e002577. doi: 10.1136/bmjresp-2024-002577, 40355297 PMC12083419

[122] Niraula P Upreti M Kadariya S Poudel B Kadariya S Kunwar S. AI/ML driven prediction of COPD exacerbations and readmissions: a systematic review and meta-analysis. Front Digit Health. (2025) 7:1641356. doi: 10.3389/fdgth.2025.1641356, 41487303 PMC12756889

[123] Chien SY Hu HC Tseng W. Design of an AI-driven home-based pulmonary telerehabilitation system to enhance patient engagement. Digit Health. (2025) 11:20552076251393295. doi: 10.1177/20552076251393295, 41229937 PMC12602929

[124] Zhang C Yu K Jin Z Bao Y Zhang C Liao J . Intelligent wearable devices with audio collection capabilities to assess chronic obstructive pulmonary disease severity. Digit Health. (2025) 11:20552076251320730. doi: 10.1177/20552076251320730, 40093702 PMC11907614

[125] Ramadurai D Lee CT Traeger L Pucci G Jackson-Sagredo A Shah S . Telehealth education leveraging electronic transitions of care for COPD patients (TELE-TOC): a study protocol for a type II hybrid effectiveness-implementation randomised, pragmatic clinical trial of a pharmacist-led intervention. BMJ Open. (2025) 15:e105521. doi: 10.1136/bmjopen-2025-105521, 41193196 PMC12587980

[126] Jiang W Jin X Du C Gu W Gao X Zhou C . Internet of things-based management versus standard management of home noninvasive ventilation in COPD patients with hypercapnic chronic respiratory failure: a multicentre randomized controlled non-inferiority trial. EClinicalMedicine. (2024) 70:102518. doi: 10.1016/j.eclinm.2024.102518, 38495520 PMC10940131

[127] Mahajan A Gilbert S. Do we need AI guardians to protect us from health information overload? NPJ Digit Med. (2025) 8:632. doi: 10.1038/s41746-025-02093-0, 41145602 PMC12559249

[128] Bergersen S Mickelson Weldingh N Westlund Hegna B Edvardsen A Lenvik O Kværner C . Effects of digital remote care on healthcare utilization and patient satisfaction in COPD [conference abstract]. Eur Respir J. (2025) 66:PA6261. doi: 10.1183/13993003.congress-2025.PA6261

[129] Ebenso B Namisango E Abejirinde IO Allsop MJ. Editorial: the scale-up and sustainability of digital health interventions in low- and middle-income settings. Front Digit Health. (2025) 7:1634223. doi: 10.3389/fdgth.2025.1634223, 41058973 PMC12497710

[130] Yew SQ Trivedi D Adanan NIH Chew BH. Facilitators and barriers to the implementation of digital health Technologies in Hospital Settings in lower- and middle-income countries since the onset of the COVID-19 pandemic: scoping review. J Med Internet Res. (2025) 27:e63482. doi: 10.2196/63482, 40053793 PMC11926458

[131] Pan Z Liao S Sun W Zhou H Lin S Chen D . Screening and early warning system for chronic obstructive pulmonary disease with obstructive sleep apnoea based on the medical internet of things in three levels of healthcare: protocol for a prospective, multicentre, observational cohort study. BMJ Open. (2024) 14:e075257. doi: 10.1136/bmjopen-2023-075257, 38418236 PMC10910414

[132] Fernandes G Williams S Adab P Gale N de Jong C de Sousa JC . Engaging stakeholders to level up COPD care in LMICs: lessons learned from the “breathe well” programme in Brazil, China, Georgia, and North Macedonia. BMC Health Serv Res. (2024) 24:66. doi: 10.1186/s12913-023-10525-4, 38216986 PMC10790249

[133] Muna ND. Bridging the evidence gap: research equity in conflict-affected health systems. Int J Equity Health. (2025) 24:293. doi: 10.1186/s12939-025-02661-6, 41146195 PMC12560315

[134] Siddharthan T Pollard SL Quaderi SA Rykiel NA Wosu AC Alupo P . Discriminative accuracy of chronic obstructive pulmonary disease screening instruments in 3 low- and middle-income country settings. JAMA. (2022) 327:151–60. doi: 10.1001/jama.2021.23065, 35015039 PMC8753498

[135] Öztürk O Kocaman R Kanbach DK. How to design bibliometric research: an overview and a framework proposal. Rev Manag Sci. (2024) 18:3333–61. doi: 10.1007/s11846-024-00738-0

