# Supplementary material for: Mobile health for chronic obstructive pulmonary disease: a bibliometric analysis based on integrated databases (2000–2025)
Source: Front Med (Lausanne). 2026 Jun 2;13:1803815. doi: 10.3389/fmed.2026.1803815 (PMC13269026; doi:10.3389/fmed.2026.1803815)
Supplement: Supplementary file 1 [file Data_Sheet_1.docx]

| Category | Specific Standard requirement |
| --- | --- |
| Citation index | Web of Science Core Collection (WoSCC); Science Citation Index Expanded (SCI-EXPANDED) |
| Topic | #1. TS= (“Chronic Obstructive Pulmonary Diseases” OR “COPD” OR “Chronic Obstructive Lung Disease” OR “Chronic Obstructive Lung Diseases” OR “Chronic Obstructive Pulmonary Disease” OR “COAD” OR “Chronic Obstructive Airway Disease” OR “Chronic Obstructive Airway Diseases” OR “Chronic Airflow Obstructions” OR “Chronic Airflow Obstruction”)  #2. TS= (“Mobile app” OR “Mobile apps” OR “Ehealth” OR “E-health” OR “Mhealth” OR “M-health” OR “Telehealth” OR “Tele-health” OR “Tele health” OR “Digital Health” OR “Digital Health Tech*” OR “Telemedicine” OR “Telecare” OR “Tele-Care” OR “Tele Care” OR “Virtual Medicine” OR “Mobile Device” OR “Mobile Devices” OR “Mobile-Based” OR “Mobile Health” OR “Mobile Healthcare” OR “Mobile Health-care” OR “Mobile Health Care" OR “Mobile Software” OR “Mobile Softwares” OR “Mobile Health Software” OR “Mobile Health Softwares” OR “Mobile Project” OR “Mobile Projects” OR “Mobile Health Project” OR “Mobile Health Projects” OR “Wearable Electronic Device” OR “Wearable Electronic Devices” OR “Wearable Electronic Tech*” OR “Wearable Devices” OR “Wearable Device” OR “Wearable Tech*” OR “Wearable Health Tech*” OR “Wearable Health Device” OR “Wearable Health Devices” OR “Electronic Skin” OR “Wearable Computer” OR “Wearable Computers” OR “Tablet Software” OR “Tablet Softwares” OR “Mobile Tablet” OR “Mobile Tablets” OR “Electronic Tablet” OR “Electronic Tablets” OR “Tablet App” OR “Tablet Apps” OR “Tablet application” OR “Tablet Applications” OR “app-based” OR “app-supported” OR “app-assisted” OR “Mobile Application” OR “Mobile Applications” OR “app” OR “apps” OR “phone-based” OR “smartphone-based” OR “tablet-based” OR “Mobile Health Application” OR “Mobile Health Applications” OR “Mobile Health App” OR “Mobile Health Apps” OR “Mobile Tech*” OR “Mobile Health Tech*” OR “Cell Phone Application” OR “Cell Phone Applications” OR “Cell Phone App” OR “Cell Phone Apps” OR “Smartphone Application” OR “Smartphone Applications” OR “Smartphone App” OR “Smartphone Apps” OR “iPhone Application” OR “iPhone Applications” OR “iPhone App” OR “iPhone Apps” OR “Android Application” OR “Android Applications” OR “Android App” OR “Android Apps” OR “Portable Electronic App” OR “Portable Electronic Apps” OR “Portable Electronic Application” OR “Portable Electronic Applications” OR “Portable Software App” OR “Portable Software Apps” OR “Portable Software Application” OR “Portable Software Applications” OR “Internet App” OR “Internet Apps” OR “Internet Application” OR “Internet Applications” OR “Web App” OR “Web Apps” OR “Web Application” OR “Web Applications” OR “Google Play” ) |
| Search strategy | #1 AND #2 |
| Document type | Article OR Review Article |
| Language | English |
| Timespan | 2000.01.01-2025.12.31 |

Supplementary Table 1. Retrieval strategy of publications in Web of Science Core Collection

Supplementary Table 2. Retrieval strategy of publications in PubMed

| Category | Specific Standard requirement |
| --- | --- |
| Research database | PubMed |
| Language | English |
| Search strategy | ( (Chronic Obstructive Pulmonary Diseases) OR (COPD) OR (Chronic Obstructive Lung Disease) OR (Chronic Obstructive Lung Diseases) OR (Chronic Obstructive Pulmonary Disease) OR (COAD) OR (Chronic Obstructive Airway Disease) OR (Chronic Obstructive Airway Diseases) OR (Airflow Obstruction, Chronic) OR (Airflow Obstructions, Chronic) OR (Chronic Airflow Obstructions) OR (Chronic Airflow Obstruction) OR (“Pulmonary Disease, Chronic Obstructive”[Mesh]) ) AND ( (“Telemedicine”[Mesh]) OR (mobile app) OR (mobile apps) OR (ehealth) OR (e-health) OR (mhealth) OR (m-health) OR (Telehealth) OR (Tele-health) OR (Tele health) OR (Digital Health) OR (Digital Health Tech*) OR (telemedicine) OR (Telecare) OR (Tele-Care) OR (Tele Care) OR (Virtual Medicine) OR (mobile device) OR (mobile devices) OR (mobile-based) OR (mobile health) OR (mobile healthcare) OR (mobile health-care) OR (mobile health care) OR (Mobile software) OR (Mobile softwares) OR (Mobile health software) OR (Mobile health softwares) OR (Mobile project) OR (Mobile projects) OR (Mobile health project) OR (Mobile health projects) OR (Wearable Electronic Device) OR (Wearable Electronic Devices) OR (Wearable Electronic Tech*) OR (Wearable Devices) OR (Wearable Device) OR (Wearable Tech*) OR (Wearable health tech*) OR (Wearable health Device) OR (Wearable health Devices) OR (Electronic Skin) OR (Wearable Computer) OR (Wearable Computers) OR (tablet software) OR (tablet softwares) OR (mobile tablet) OR (mobile tablets) OR (electronic tablet) OR (electronic tablets) OR (Tablet App) OR (Tablet Apps) OR (Tablet application) OR (Tablet applications) OR (app-supported) OR (app-assisted) OR (mobile application) OR (mobile applications) OR (app) OR (apps) OR (app-based) OR (phone-based) OR (smartphone-based) OR (tablet-based) OR (Mobile health application) OR (Mobile health applications) OR (Mobile health app) OR (Mobile health apps) OR (Mobile tech*) OR (Mobile health tech*) OR (Cell phone application) OR (Cell phone applications) OR (Cell phone app) OR (Cell phone apps) OR (Smartphone application) OR (Smartphone applications) OR (Smartphone app) OR (Smartphone apps) OR (iPhone application) OR (iPhone applications) OR (iPhone app) OR (iPhone apps) OR (Android application) OR (Android applications) OR (Android app) OR (Android apps) OR (Portable Electronic App) OR (Portable Electronic Apps) OR (Portable Electronic Application) OR (Portable Electronic Applications) OR (Portable Software App) OR (Portable Software Apps) OR (Portable Software Application) OR (Portable Software Applications) OR (internet app) OR (internet apps) OR (internet application) OR (internet applications) OR (web app) OR (web apps) OR (web application) OR (web applications) OR (Google Play) OR (App store) ) AND ((clinicaltrial[Filter] OR randomizedcontrolledtrial[Filter]) AND (english[Filter]) AND (2000:2025[pdat])) |

Supplementary Table 3. The BIBLIO checklist for reporting bibliometric reviews of the biomedical literature

| **Section/Topic** | **Item**  **No.** | **Checklist item** | **Reported**  **on page No.** |
| --- | --- | --- | --- |
| **Title** |  |  |  |
| Identification | 1 | Identify the report as a bibliometric review in the title. | 1 |
| Issues/topics | 2 | Indicate the key issues/topics under investigation and coverage of time period. | 1 |
| **Abstract** |  |  |  |
| Structured summary | 3 | Structured summary including (as applicable): background, methods, results (key findings) and conclusions. | 1, 2, 3 |
| **Introduction/Background** |  |  |  |
| Justification/Rationale/Explanation | 4 | Present review of existing knowledge and  epidemiological information. | 4, 5, 6 |
| Objectives | 5 | Statement of the objective (s) or question (s). | 6 |
| **Methods** |  |  |  |
| Search engines (data sources) | 6 | Describe all information sources (such as electronic  databases, contact with study authors, trial registers or other grey literature sources). | 6, 7 |
| Search strategy | 7 | Keywords and systematization criteria (date of search, language, type of document) for the search. | 7 |
| Time period | 8 | The period that the review covers and the justification. | 7 |
| Eligibility criteria | 9 | Describe all inclusion and exclusion criteria; languages; study design, type of publication and time period. | 7, 8, 9 |
| Data refinement (data selection procedure) | 10 | Remove the irrelevant articles; inspection to eliminate duplicate and unrelated articles (after evaluation of the title, abstract and content). | 7, 8, 9 |
| Quality assessment (optional) | 11 | Assessment of papers by three authors and the use of assessing checklists. | 7, 8 |
| Data synthesis | 12 | Describe the methods used for summarizing, handling, synthesis, tabulations or schematic displays. Describe how the data were analysed. | 9, 10 |
| **Results** |  |  |  |
| Descriptive findings (statistics) | 13 | -Provide details of the search and selection process in a flow diagram.  -Number of citations retrieved (number of publication, year of publication, types of documents, country of  publication, articles with the highest impact, most  impactful authors, most impactful articles, authors with the highest production, top journals, top institutions, …) | 10–29 |
| Schematic map and trend | 14 | Summarize and/or present the schematic maps and trends using appropriate software to present citations,  journals, authors, top journals, time trends, emerging  literature, and any relevant indicators (as applicable)^[1-5].^ | 10–29 |
| Tabulation and summarizing the findings | 15 | General recommendation: Studies under consideration could be summarized and organized by different subtitles and different scenarios. Regardless, results need to be  presented in separate tables covering each subtitle. The following are some options that could help to summarize the findings.  *Option 1:*  -Start the presentation with a historical view [when and who first published on the topic].  -Report on review papers. The result should be listed in a separate table. Also, specify the review type (scoping  review, narrative review, systematic review, and meta-analysis).  -Summarize the findings according to the study designs and main study types.  *Option 2:*  -Start the presentation with a historical view [when and who first published on the topic].  -Report on review papers. The result should be listed in a separate table. Also, indicate the review type (scoping  review, narrative review, systematic review, and meta-analysis) should be specified.  -Summarize the findings according to outcome measures or populations. For example, see^[6]^.  *Option 3:*  -Start the presentation with a historical view [when and who first published on the topic]. | 10–29 |
|  |  | -Report on review papers. The result should be listed in a separate table. Also, specify the review type (scoping  review, narrative review, systematic review, and meta- analysis).  -Summarize the findings according to concept^[7]^.  *Option 4:*  -Start the presentation with a historical view [when and who first published on the topic].  -Report on review papers. The result should be listed in a separate table, and also specify the review type (scoping review, narrative review, systematic review, and meta-analysis).  -Summarize the findings according to different subtitles relevant to the main topic^[8]^. | 10–29 |
| Synthesis of findings | 16 | Synthesize the findings as much as possible, find the gap, and propose a model, hypothesis, etc. (if applicable). | 10–29 |
| **Discussion** |  |  |  |
| Summary of evidence | 17 | Summarize the main findings. The findings should be presented in more “general” or “accessible” terms. | 30–42 |
| Interpretation | 18 | Include interpretation consistent with results.  Explanations for observed outcomes, similarities, and differences reported would be essential. | 30–42 |
| Strengths and limitations | 19 | Discuss the strengths and limitations. | 39–41 |
| Conclusion(s) | 20 | Provide a general interpretation of the results with respect to the review questions and objectives, as well as  potential implications. | 41–42 |
| 1. McDougal L, Dehingia N, Cheung WW, Dixit A, Raj A. COVID-19 burden, author affiliation and Women’s well-being: A bibliometric analysis of COVID-19 related publications including focus on low-and middle-income countries. eClinicalMedicine 2022; 52: 101606.  2. Henstock L, Wong R, Tsuchiya A, Spencer A. Behavioral theories that have influenced the way health state preferences are elicited and interpreted: A bibliometric mapping analysis of the time trade-off method with VOSviewer visualization. Front Health Serv 2022; 2: 848087.  3. Bodea F, Bungau SG, Negru AP, Radu A, Tarce AG, Tit DM, et al. Exploring new therapeutic avenues for ophthalmic disorders: Glaucoma-related molecular docking evaluation and bibliometric analysis for improved management of ocular diseases. Bioengineering 2023; 10(8): 983.  4. Sang XZ, Wang CQ, Chen W, Rong H, Hou LJ. An exhaustive analysis of post-traumatic brain injury dementia using bibliometric methodologies. Front Neurol 2023; 14: 1165059.  5. Ramli MI, Hamzaid NA, Engkasan JP, Usman J. Respiratory muscle training: a bibliometric analysis of 60 years’ multidisciplinary journey. Biomed Eng Online 2023; 22(1): 50.  6. Akosman I, Kumar N, Mortenson R, Lans A, De La Garza Ramos R, Eleswarapu A, et al. Racial differences in perioperative complications, readmissions, and mortalities after elective spine surgery in the United States: A systematic review using AI-assisted bibliometric analysis. Glob Spine J 2023: 21925682231186759.  7. Tavousi M, Mohammadi S, Sadighi J, Zarei F, Kermani RM, Rostami R, Montazeri A. Measuring health literacy: A systematic review and bibliometric analysis of instruments from 1993 to 2021. PLOS ONE 2022; 17(7): e0271524.  8. Montazeri A. Health-related quality of life in breast cancer patients: A bibliographic review of the literature from 1974 to 2007. J Exp Clin Cancer Res 2008; 27: 32. | | | |

**Rights and permissions:** The original source of the checklist is: Montazeri A, Mohammadi S, M. Hesari P, Ghaemi M, Riazi H, Sheikhi‑Mobarakeh Z. Preliminary guideline for reporting bibliometric reviews of the biomedical literature (BIBLIO): a minimum requirement. *Systematic Reviews* 2023; 12: 239. doi.org/10.1186/s13643-023-02410-2. The article is licensed under a Creative Commons Attribution 4.0 International License (<http://creativecommons.org/licenses/by/4.0/>). A change was made to the original checklist to add full references to the cited sources.
